# Supplementary figures and images for: Composition of nitrogen in urban residential stormwater runoff: Concentrations, loads, and source characterization of nitrate and organic nitrogen
Source: PLoS One. 2020 Feb 28;15(2):e0229715. doi: 10.1371/journal.pone.0229715 (PMC7048309; doi:10.1371/journal.pone.0229715)

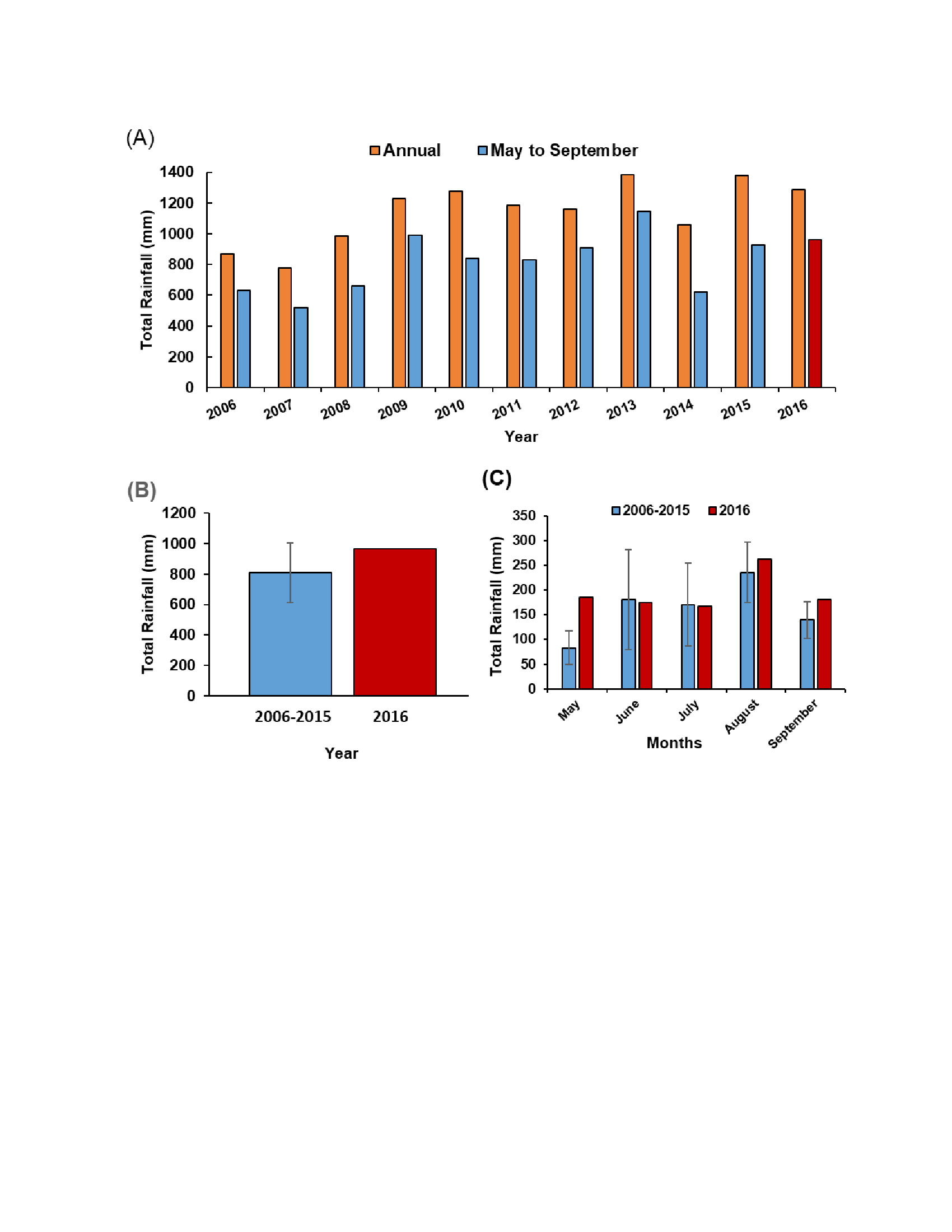

Supplement: S1 Fig — (A) Annual and May to September rainfall from 2006 to 2016, (B) comparison of mean 10 years (2006–2015) and 2016 rainfall, and (C) comparison of monthly rainfall from May to September for 2006–2015 and 2016 in the study site located in Bradenton, Florida. Red bar in (A) indicates data of sampling year (2016). (TIFF) [file pone.0229715.s001.tiff]

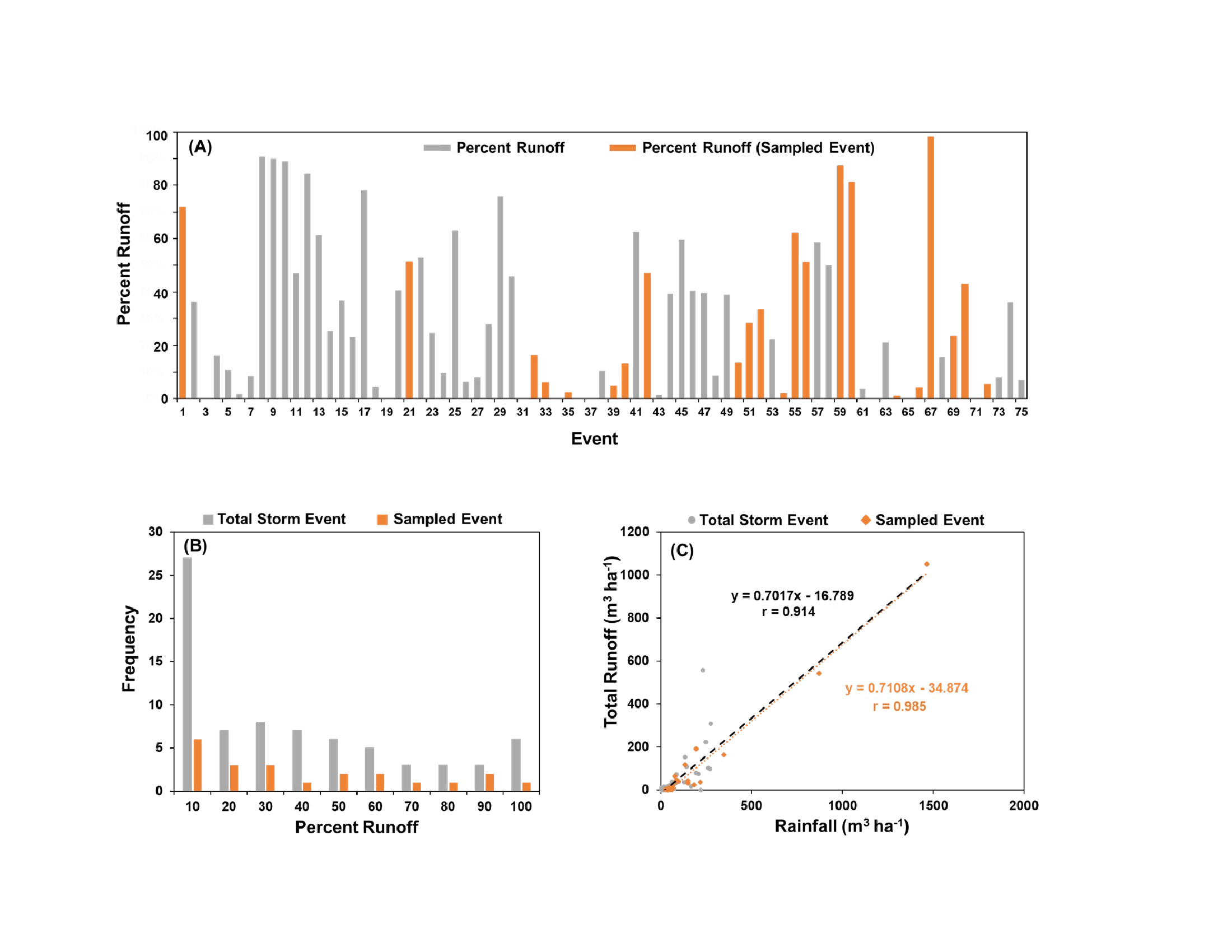

Supplement: S2 Fig — (A) Percent runoff of total rainfall, (B) frequency distribution of percent runoff, and (C) relationship between rainfall and runoff amount for total 75 storm events and sampled 22 storm events from May to September, 2016 (line fits for both 75 and 22 events are shown). (TIFF) [file pone.0229715.s002.tiff]

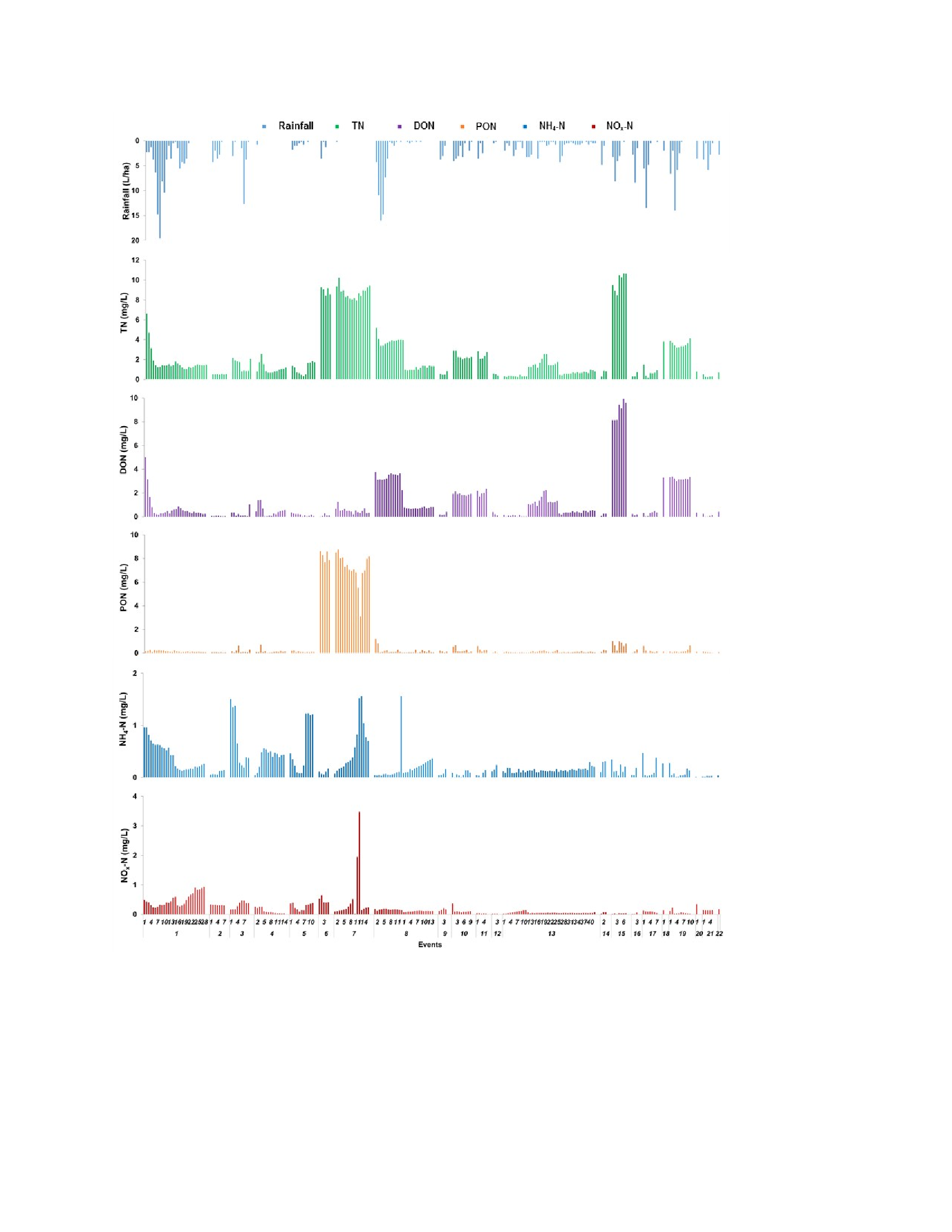

Supplement: S3 Fig — (TIFF) [file pone.0229715.s003.tiff]

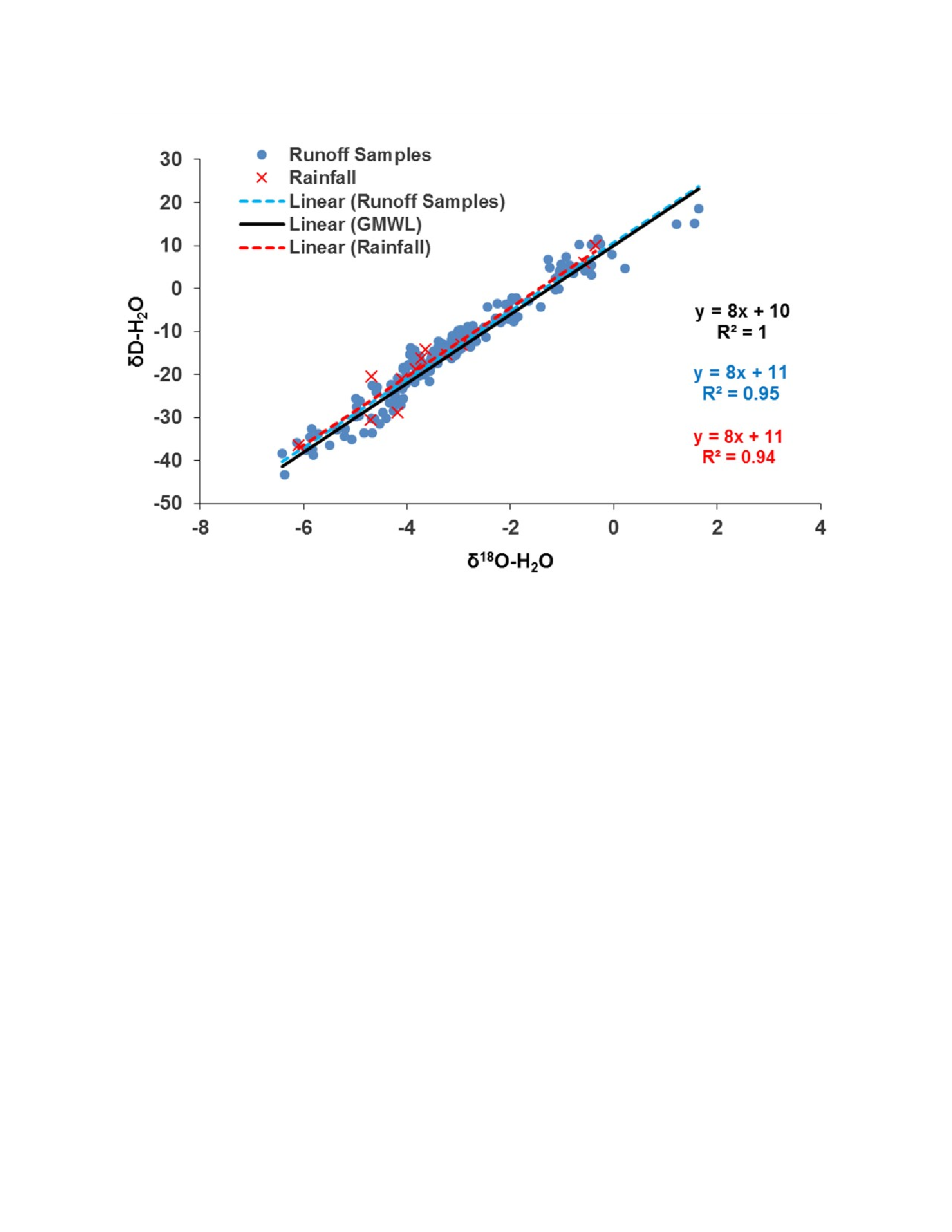

Supplement: S4 Fig — (TIFF) [file pone.0229715.s004.tiff]

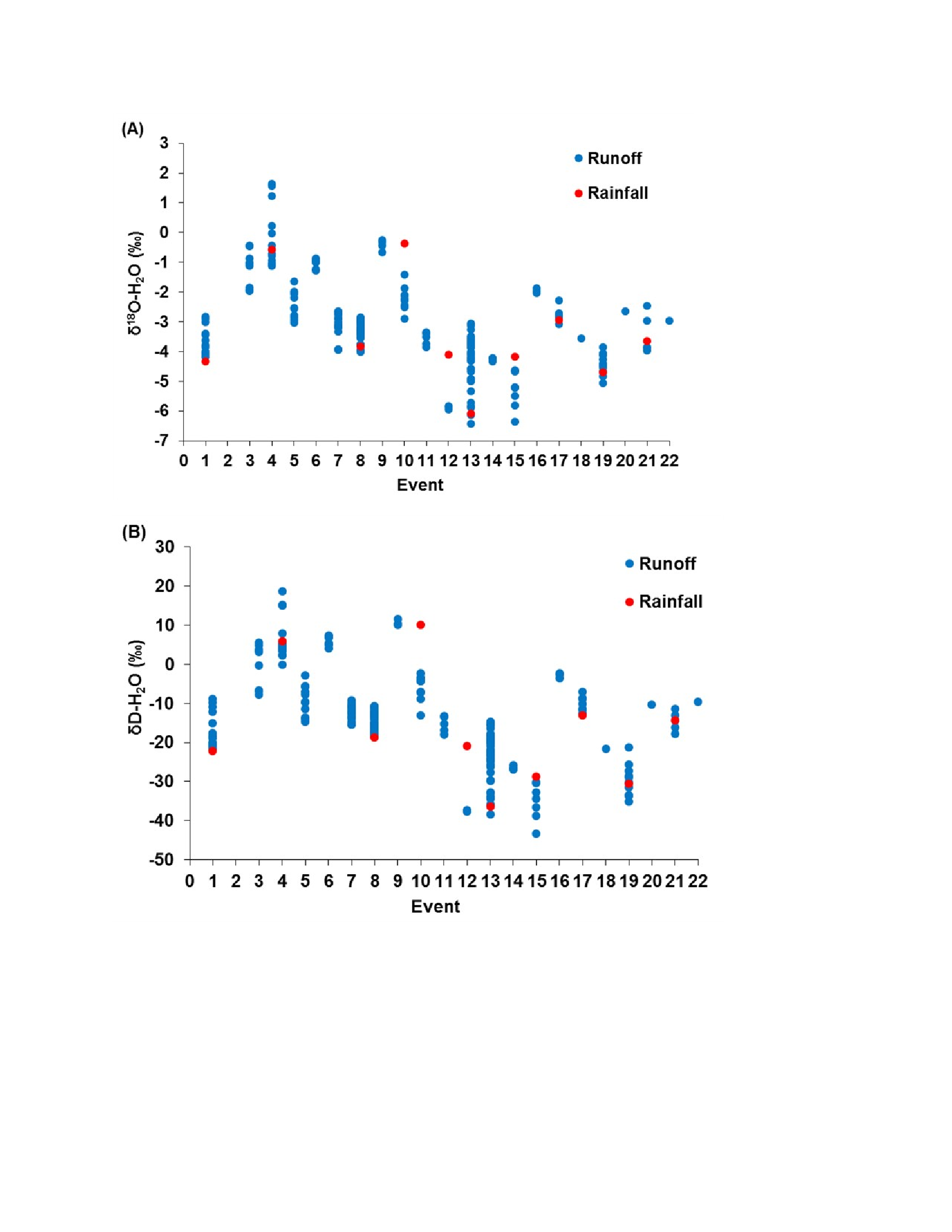

Supplement: S5 Fig — Variation in stable isotope composition (A) δ18O–H2O and (B) δD–H2O in rainfall (n = 10) and stormwater runoff samples (n = 176) collected during 22 storm events from May to September, 2016. (TIFF) [file pone.0229715.s005.tiff]

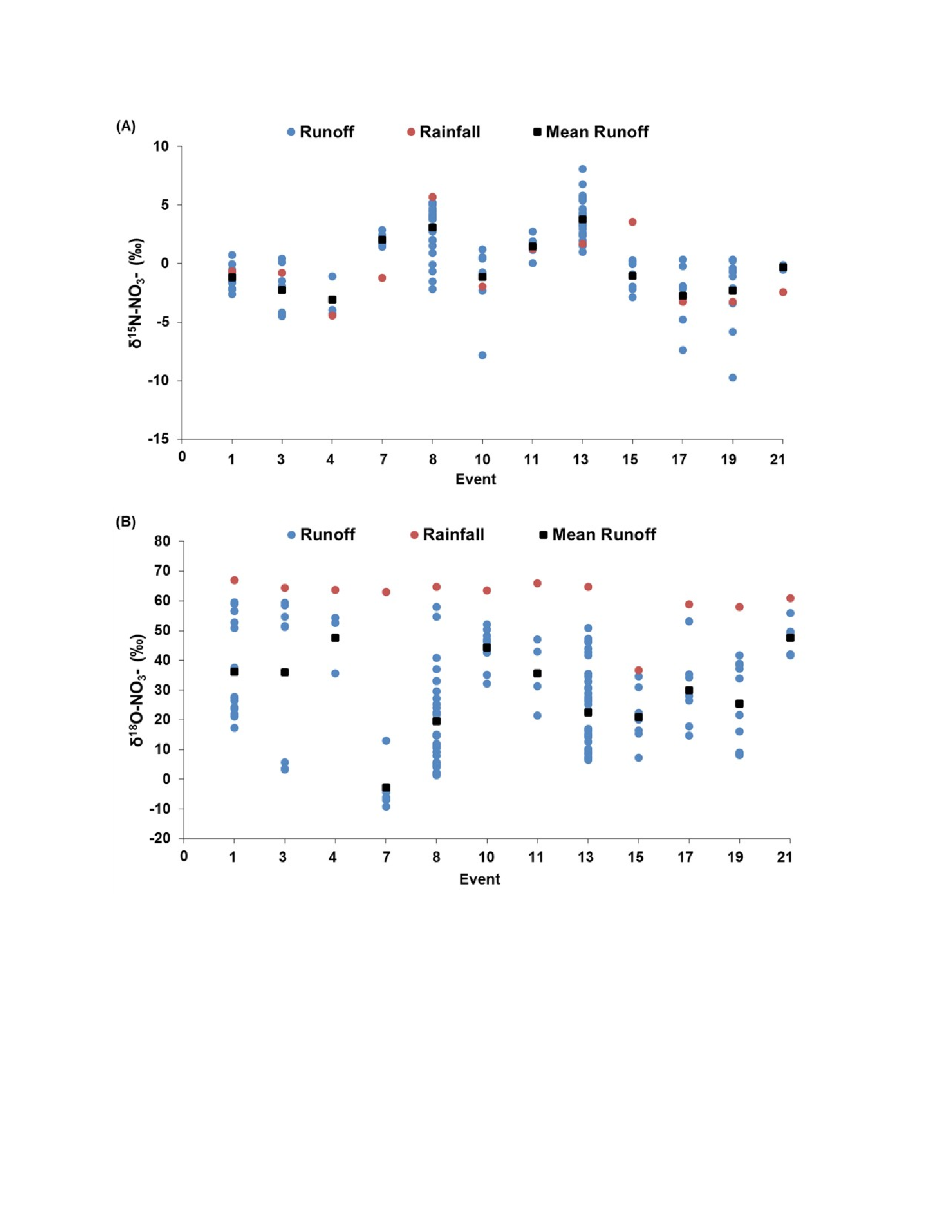

Supplement: S6 Fig — Variation in stable isotope composition (A) δ15N-NO3- and (B) δ18O-NO3- in rainfall (n = 12) and stormwater runoff samples collected during 22 storm events from May to September, 2016. (TIFF) [file pone.0229715.s006.tiff]
