# Supplementary material for: Composition of nitrogen in urban residential stormwater runoff: Concentrations, loads, and source characterization of nitrate and organic nitrogen
Source: PLoS One. 2020 Feb 28;15(2):e0229715. doi: 10.1371/journal.pone.0229715 (PMC7048309; doi:10.1371/journal.pone.0229715)
Supplement: S1 Table — (PDF) [file pone.0229715.s007.pdf]

S1 Table. Pervious and impervious area of residential catchment located in Lakewood Ranch, Bradenton, Florida, United States.

	**Area (hectares)**	**Percent (%)**
**Pervious:**		57.10
**Canopy**	0.24	6.13
**Grass**	1.98	50.96
**Impervious:**		42.90
**Houses**	0.72	18.45
**Roads**	0.95	24.45
**Total**	3.89	100
