# Supplementary material for: Composition of nitrogen in urban residential stormwater runoff: Concentrations, loads, and source characterization of nitrate and organic nitrogen
Source: PLoS One. 2020 Feb 28;15(2):e0229715. doi: 10.1371/journal.pone.0229715 (PMC7048309; doi:10.1371/journal.pone.0229715)
Supplement: S2 Table — (PDF) [file pone.0229715.s008.pdf]

S2 Table. End-member literature values of $\delta^{18}O–NO_3^-$ and $\delta^{15}N–NO_3^-$.

Sources	$\delta^{18}O–NO_3^-$ (‰)	$\delta^{15}N–NO_3^-$ (‰)	References
**a) Atmospheric deposition**	+23 to +75 ($AgNO_3$ method)	-15 to +15	Kendall et al. (2007); Yang and Toor (2016)
**- Fossil fuel combustion**	–	+3.4 to +17	Felix et al. (2015)
**- Lightning**	–	-0.5 to +1.4	Hoering (1957)
**- Coal-fired power plant**	–	+4.8 to +13	Heaton (1990); Felix et al. (2012)
**b) NO_3^- fertilizer**	+17 to +25	-5 to +10	Kendall et al. (2007); Yang and Toor (2016)
**c) NH_4^+ fertilizer**	-15 to +15	-10 to +5	
**d) Organic N**	-15 to +15	0 to +25	
**e) Soil NH_4^+**	-15 to +15	+2 to +8	
