# Supplementary material for: Composition of nitrogen in urban residential stormwater runoff: Concentrations, loads, and source characterization of nitrate and organic nitrogen
Source: PLoS One. 2020 Feb 28;15(2):e0229715. doi: 10.1371/journal.pone.0229715 (PMC7048309; doi:10.1371/journal.pone.0229715)
Supplement: S3 Table — (PDF) [file pone.0229715.s009.pdf]

S3 Table. ^{13}C and ^{15}N of PON in various landscape sources and collected stormwater runoff samples.

Sources	Collection Date	$\delta^{13}C$ (‰)	$\delta^{15}N$ (‰)
**St. Augustine grass (n=21)**	April 2016	-17.80 to -11.62 (mean: -14.21±1.590)	-1.93 to +0.68 (mean: -0.46±0.785)
**Acorns (n=3)**	April 2016	-30.79 to -28.44 (mean: -29.39±1.238)	1.55 to 1.60 (mean: 1.58±0.026)
**Oak leaves (n=3)**	April 2016	-29.90 to -27.41 (mean: -28.84±1.284)	-1.70 to -0.83 (mean: -1.24±0.436)
**Runoff samples**	May to September 2016	-28.31 to -19.46 (mean: -23.04±1.725)	-1.99 to 6.27 (mean: 1.03±1.384)
