# Supplementary material for: Composition of nitrogen in urban residential stormwater runoff: Concentrations, loads, and source characterization of nitrate and organic nitrogen
Source: PLoS One. 2020 Feb 28;15(2):e0229715. doi: 10.1371/journal.pone.0229715 (PMC7048309; doi:10.1371/journal.pone.0229715)
Supplement: S4 Table — (PDF) [file pone.0229715.s010.pdf]

S4 Table. Runoff volume variables, flow-weighted mean concentration of nitrogen forms, and $\delta^{18}O–NO_3^-$ and $\delta^{15}N–NO_3^-$ values for 22 storm events from May to September, 2016.

Date	Event	No. of Samples	Flow-Weighted Mean Concentration (mg/L)					Range (mean)	
			TN	NH_3-N	NO_x-N	DON	PON	$\delta^{18}O–NO_3^-$ (‰)	$\delta^{15}N–NO_3^-$ (‰)
**5/4/2016**	1	28	1.39	0.45	0.33	0.46	0.16	17.29 – 59.70 (36.15)	-2.62 – 0.74 (-1.15)
**6/28/2016**	2	7	0.52	0.07	0.32	0.07	0.07		
**7/27/2016**	3	9	1.15	0.48	0.39	0.17	0.10	3.34 – 59.47 (36.04)	-4.48 – 0.45 (-2.24)
**7/29/2016**	4	14	1.20	0.37	0.14	0.56	0.14	35.68 – 54.38 (47.53)	-4.25 – 1.07 (-3.10)
**7/31/2016**	5	11	1.19	0.43	0.36	0.22	0.18		
**8/7/2016**	6	5	9.01	0.08	0.56	0.09	8.28		
**8/8/2016**	7	16	8.90	0.31	0.34	0.58	7.67	-9.19 – 12.91 (-2.74)	1.45 – 2.88 (2.05)
**8/10/2016**	8	27	3.54	0.06	0.16	3.11	0.19	1.37 – 58.04 (19.55)	-2.15 – 5.23 (3.06)
**8/20/2016**	9	4	0.50	0.05	0.15	0.16	0.13		
**8/26/2016**	10	9	2.23	0.09	0.09	1.89	0.16	32.10 – 52.08 (44.36)	-7.84 – 1.20 (-1.14)
**8/27/2016**	11	5	2.66	0.04	0.04	2.12	0.47	21.44 – 47.07 (35.67)	0.04 – 2.73 (1.46)
**8/30/2016**	12	3	0.54	0.14	0.02	0.31	0.07		
**8/31/2016**	13	42	0.68	0.13	0.05	0.43	0.07	6.64 – 50.84 (22.45)	0.99 – 8.06 (3.77)
**9/1/2016**	14	3	0.58	0.20	0.05	0.16	0.18		

S4 Table. Continued

Date	Event	No. of Samples	Flow-Weighted Mean Concentration (mg/L)					Range (mean)	
			TN	NH_3-N	NO_x-N	DON	PON	$\delta^{18}O–NO_3^-$ (‰)	$\delta^{15}N–NO_3^-$ (‰)
**9/5/2016**	15	7	10.11	0.06	0.03	9.17	0.85	7.29 – 34.63 (21.00)	-2.85 – 0.32 (-1.06)
**9/8/2016**	16	3	0.45	0.09	0.02	0.15	0.19		
**9/12/2016**	17	7	0.43	0.06	0.09	0.14	0.13	14.64 – 53.13 (29.98)	-7.39 – 0.36 (-2.73)
**9/16/2016**	18	1	3.82	0.27	0.13	3.29	0.13		
**9/18/2016**	19	10	3.26	0.03	0.03	3.10	0.10	8.20 – 41.76 (25.37)	-9.72 – 0.34 (-2.32)
**9/24/2016**	20	1	0.80	0.01	0.35	0.34	0.11		
**9/25/2016**	21	5	0.28	0.03	0.13	0.07	0.06	41.68 – 55.85 (47.61)	-0.50 – -0.11 (-0.29)
**9/27/2016**	22	1	0.69	0.04	0.18	0.40	0.07		
