# Supplementary material for: Composition of nitrogen in urban residential stormwater runoff: Concentrations, loads, and source characterization of nitrate and organic nitrogen
Source: PLoS One. 2020 Feb 28;15(2):e0229715. doi: 10.1371/journal.pone.0229715 (PMC7048309; doi:10.1371/journal.pone.0229715)
Supplement: S5 Table — (PDF) [file pone.0229715.s011.pdf]

S5 Table. Pearson correlation among rainfall variables and nitrogen forms from May to September, 2016.

Forms (g)	Total Rainfall	Duration	Intensity	Antecedent Dry Period
**TN**	0.69**	ns	0.52*	0.47*
**DON**	ns	ns	0.50*	ns
**PON**	0.73**	0.55**	ns	0.67*
**NH_4-N**	0.90**	0.45*	ns	0.94**
**NO_x-N**	0.90**	0.47*	ns	0.94**
**DIN**	0.90 **	0.46*	ns	0.94**

* Correlation is significant at $p<0.05$

** Correlation is significant at $p<0.001$

ns= not significant
