# Supplementary material for: Composition of nitrogen in urban residential stormwater runoff: Concentrations, loads, and source characterization of nitrate and organic nitrogen
Source: PLoS One. 2020 Feb 28;15(2):e0229715. doi: 10.1371/journal.pone.0229715 (PMC7048309; doi:10.1371/journal.pone.0229715)
Supplement: Attachment — Submitted filename: PONE-D-19-21012_reviewer.pdf [file pone.0229715.s012.pdf]

PLOS ONE

Composition of Nitrogen in Urban Residential Stormwater Runoff: Variation in Concentrations, Loads, and Source Characterization of Nitrate and Organic Nitrogen

--Manuscript Draft--

**Manuscript Number:**	PONE-D-19-21012
**Article Type:**	Research Article
**Full Title:**	Composition of Nitrogen in Urban Residential Stormwater Runoff: Variation in Concentrations, Loads, and Source Characterization of Nitrate and Organic Nitrogen
**Short Title:**	Nitrogen in urban stormwater runoff
**Corresponding Author:**	Gurpal S. Toor, Ph.D.<br>University of Maryland<br>College Park, Maryland UNITED STATES
**Keywords:**	Nitrogen; Stable isotopes; Dissolved organic nitrogen; Stormwater runoff
**Abstract:**	Stormwater runoff is a leading cause of nitrogen (N) transport to water bodies and hence one means of water quality deterioration. Stormwater runoff was monitored in an urban residential catchment (drainage area: 3.89 hectares) in Florida, United States to investigate the concentrations, forms, and sources of N. Runoff samples were collected from a residential stormwater pipe over 22 storm events during May–September 2016. Collected runoff samples were analyzed for various N forms such as ammonium (NH4–N), nitrate (NOx–N), dissolved organic nitrogen (DON), and particulate organic nitrogen (PON), and isotopic characterization tools were used to infer sources of NO3–N and PON. The DON was the dominant N form in runoff (47%) followed by PON (22%), NOx–N (17%), and NH4–N (14%). NOx–N, NH4–N and PON were positively correlated with total rainfall and antecedent dry period, suggesting longer dry periods and higher rainfall amounts are significant drivers for transport of these N forms. Whereas DON was positively correlated to only rainfall intensity indicating that higher intensity rain may flush out DON from soils and/or cause leaching of DON from particulates. Stable isotopes of NO3– showed a shifting pattern of NO3– sources from atmospheric deposition to inorganic N fertilizers in events with higher and longer duration of rainfall. The stable isotopes of PON confirmed that plant material on impervious surfaces (oak detritus, grass clippings) were the main sources of PON in stormwater runoff. Our results demonstrate that practices targeting both inorganic and organic N are needed to control N transport from residential catchments to receiving waters.
**Order of Authors:**	Jariani Jani
	Mary Lusk
	Yun-Ya Yang
	Gurpal S. Toor, Ph.D.
**Additional Information:**	
**Question**	**Response**
**Financial Disclosure**<br><br>Enter a financial disclosure statement that describes the sources of funding for the work included in this submission. Review the submission guidelines for detailed requirements. View published research articles from *PLOS ONE* for specific examples.<br><br>This statement is required for submission	GST received funding from Florida Department of Environmental Protection. There is no grant number. The funders had no role in study design, data collection and analysis, decision to publish, or preparation of the manuscript.

Powered by Editorial Manager® and ProduXion Manager® from Aries Systems Corporation

and **will appear in the published article** if the submission is accepted. Please make sure it is accurate.<br><br>**Unfunded studies**<br>Enter: *The author(s) received no specific funding for this work.*<br><br>**Funded studies**<br>Enter a statement with the following details:<br>• Initials of the authors who received each award<br>• Grant numbers awarded to each author<br>• The full name of each funder<br>• URL of each funder website<br>• Did the sponsors or funders play any role in the study design, data collection and analysis, decision to publish, or preparation of the manuscript?<br>• **NO** - Include this sentence at the end of your statement: *The funders had no role in study design, data collection and analysis, decision to publish, or preparation of the manuscript.*<br>• **YES** - Specify the role(s) played.<br><br>* typeset	
**Competing Interests**<br><br>Use the instructions below to enter a competing interest statement for this submission. On behalf of all authors, disclose any competing interests that could be perceived to bias this work—acknowledging all financial support and any other relevant financial or non-financial competing interests.<br><br>This statement **will appear in the published article** if the submission is accepted. Please make sure it is accurate. View published research articles from *PLOS ONE* for specific examples.	he authors have declared that no competing interests exist.

**NO authors have competing interests**<br><br>Enter: *The authors have declared that no competing interests exist.*<br><br>**Authors with competing interests**<br><br>Enter competing interest details beginning with this statement:<br><br>*I have read the journal's policy and the authors of this manuscript have the following competing interests: [insert competing interests here]*<br><br>* typeset	
**Ethics Statement**<br><br>Enter an ethics statement for this submission. This statement is required if the study involved:<br><br>• Human participants<br>• Human specimens or tissue<br>• Vertebrate animals or cephalopods<br>• Vertebrate embryos or tissues<br>• Field research<br><br>Write "N/A" if the submission does not require an ethics statement.<br><br>General guidance is provided below. Consult the submission guidelines for detailed instructions. **Make sure that all information entered here is included in the Methods section of the manuscript.**	N/A

Powered by Editorial Manager® and ProduXion Manager® from Aries Systems Corporation

**Format for specific study types**<br><br>**Human Subject Research (involving human participants and/or tissue)**<br>• Give the name of the institutional review board or ethics committee that approved the study<br>• Include the approval number and/or a statement indicating approval of this research<br>• Indicate the form of consent obtained (written/oral) or the reason that consent was not obtained (e.g. the data were analyzed anonymously)<br><br>**Animal Research (involving vertebrate animals, embryos or tissues)**<br>• Provide the name of the Institutional Animal Care and Use Committee (IACUC) or other relevant ethics board that reviewed the study protocol, and indicate whether they approved this research or granted a formal waiver of ethical approval<br>• Include an approval number if one was obtained<br>• If the study involved *non-human primates*, add *additional details* about animal welfare and steps taken to ameliorate suffering<br>• If anesthesia, euthanasia, or any kind of animal sacrifice is part of the study, include briefly which substances and/or methods were applied<br><br>**Field Research**<br><br>Include the following details if this study involves the collection of plant, animal, or other materials from a natural setting:<br>• Field permit number<br>• Name of the institution or relevant body that granted permission	
**Data Availability**<br><br>Authors are required to make all data underlying the findings described fully available, without restriction, and from the time of publication. PLOS allows rare exceptions to address legal and ethical concerns. See the PLOS Data Policy and FAQ for detailed information.	Yes - all data are fully available without restriction

A Data Availability Statement describing where the data can be found is required at submission. Your answers to this question constitute the Data Availability Statement and **will be published in the article**, if accepted.<br><br>**Important:** Stating 'data available on request from the author' is not sufficient. If your data are only available upon request, select 'No' for the first question and explain your exceptional situation in the text box.<br><br>Do the authors confirm that all data underlying the findings described in their manuscript are fully available without restriction?	
**Describe where the data may be found in full sentences. If you are copying our sample text, replace any instances of XXX with the appropriate details.**<br><br>• If the data are **held or will be held in a public repository**, include URLs, accession numbers or DOIs. If this information will only be available after acceptance, indicate this by ticking the box below. For example: *All XXX files are available from the XXX database (accession number(s) XXX, XXX.).*<br>• If the data are all contained **within the manuscript and/or Supporting Information files**, enter the following: *All relevant data are within the manuscript and its Supporting Information files.*<br>• If neither of these applies but you are able to provide **details of access elsewhere**, with or without limitations, please do so. For example:<br><br>*Data cannot be shared publicly because of [XXX]. Data are available from the XXX Institutional Data Access / Ethics Committee (contact via XXX) for researchers who meet the criteria for access to confidential data.*<br><br>*The data underlying the results presented in the study are available from (include the name of the third party*	All relevant data are within the manuscript and its Supporting Information files.

and contact information or URL).<br>• This text is appropriate if the data are owned by a third party and authors do not have permission to share the data.<br><br>* typeset	
Additional data availability information:	

Powered by Editorial Manager® and ProduXion Manager® from Aries Systems Corporation

Composition of Nitrogen in Urban Residential Stormwater Runoff: Variation in Concentrations, Loads, and Source Characterization of Nitrate and Organic Nitrogen

Short title: ***Nitrogen in urban stormwater runoff***

Jariani Jani[1], Mary G. Lusk[2], Yun-Ya Yang[3], and Gurpal S. Toor[3*]

[1] Chemistry Department, Faculty of Science, University of Malaya, 50603 Kuala Lumpur, Malaysia.

[2] Gulf Coast Research and Education Center, University of Florida, Wimauma, FL 33598, USA

[3] Department of Environmental Science and Technology, University of Maryland, College Park, MD 20742, USA

* Corresponding author (gstoor@umd.edu)

ABSTRACT

Stormwater runoff is a leading cause of nitrogen (N) transport to water bodies and hence one means of water quality deterioration. Stormwater runoff was monitored in an urban residential catchment (drainage area: 3.89 hectares) in Florida, United States to investigate the concentrations, forms, and sources of N. Runoff samples were collected from a residential stormwater pipe over 22 storm events during May–September 2016. Collected runoff samples were analyzed for various N forms such as ammonium (NH_4–N), nitrate (NO_x–N), dissolved organic nitrogen (DON), and particulate organic nitrogen (PON), and isotopic characterization tools were used to infer sources of NO_3–N and PON. The DON was the dominant N form in runoff (47%) followed by PON (22%), NO_x–N (17%), and NH_4–N (14%). NO_x–N, NH_4–N and PON were positively correlated with total rainfall and antecedent dry period, suggesting longer dry periods and higher rainfall amounts are significant drivers for transport of these N forms. Whereas DON was positively correlated to only rainfall intensity indicating that higher intensity rain may flush out DON from soils and/or cause leaching of DON from particulates. Stable isotopes of NO_3^- showed a shifting pattern of NO_3^- sources from atmospheric deposition to inorganic N fertilizers in events with higher and longer duration of rainfall. The stable isotopes of PON confirmed that plant material on impervious surfaces (oak detritus, grass clippings) were the main sources of PON in stormwater runoff. Our results demonstrate that practices targeting both inorganic and organic N are needed to control N transport from residential catchments to receiving waters.

Keywords

Nitrogen; Stable isotopes; Dissolved organic nitrogen; Stormwater

Introduction

Urbanization and anthropogenic activities have accelerated nutrient enrichment and water quality problems in urban waters [1, 2]. Nitrogen (N) is often a limiting nutrient in coastal waters [3-5], where excess N loading can lead to cultural eutrophication and algal proliferation [6] . Stormwater runoff is one transport vector of N from urban areas to receiving water bodies and thus a critical source to consider in finding strategies to reduce N enrichment of urban waters [7].

Nitrogen loading to urban waters via stormwater occurs in several forms, including inorganic forms i.e. ammonium (NH_4^+), nitrite (NO_2^-) and nitrate (NO_3^-) or organic forms i.e. dissolved organic N (DON) and particulate organic N (PON). Recent studies have shown that organic N can be a large proportion of N in urban stormwater, streams, estuaries, and that portions of the DON pool can be bioavailable to the organisms that cause harmful algal blooms [6, 8-10]. The potential high proportion and bioavailability of DON indicate the need to shift from traditional stormwater management practices that focus on inorganic N to new efforts that focus on DON [9, 11]. Thus, we need better understanding of composition of N in urban waters, including information on the contribution of organic N in stormwater runoff. To date, only a handful of studies have investigated DON in urban stormwater runoff and even fewer studies have considered PON in stormwater [10, 11].

The transport of N—both organic and inorganic—via urban stormwater runoff may be altered by changes in hydrology in watersheds [12, 13]. Changes in hydrology can be related to weather (e.g., rainfall, temperature) and/or land use changes (e.g., urbanization, dam and reservoir release), which influence the hydrological pathways [14-16]. Investigation of hydrologic trends and variability of N forms is useful to determine hydrologic variables that play important roles in N transport and to elucidate potential influences of rainfall patterns on N form-

specific transport mechanisms. Rainfall variables such as rainfall amount, duration, intensity, flow, and antecedent dry periods have been used to determine the relationship between rainfall variables to nutrient and/or pollutant transport [17-19]. For example, a study by Schiff et al. [20] on stormwater runoff from parking lots in California, US showed that 18 measured compounds were inversely correlated to rainfall duration where longer rain events decreased the concentrations of the constituents in parking lot runoff. Liu et al. [21] found that TN concentration in runoff waters from an N-fertilized field was significantly correlated with rainfall amount as more rainfall generated more N runoff. Antecedent dry period was also reported to be a factor affecting nutrient transport in urban land where greater NH_4–N concentrations were observed after a long dry season [22].

In addition to considering all N forms, including organic forms, and relating their transport to rainfall variables, it is also important to elucidate sources of N to watersheds. Sources of N in urban landscapes include atmospheric deposition, anthropogenic (e.g., fertilizers, automotive detergent, pet waste), and organic materials (e.g., throughfall from the urban tree canopy, leaf litter, grass clippings) [12, 13, 23, 24]. A study in Pittsburgh, US showed that NO_3–N sources in an urban stream included atmospheric deposition (6 to 34%), sewage (72 to 94%), and denitrification processes (7 to 22%) [24]. A study by Hobbie et al. [25] on stormwater runoff in St. Paul, Minnesota, US showed that N sources in the catchment included atmospheric deposition (19 to 34%), chemical fertilizers (37 to 59%), and pet waste (28%). Studies conducted in Tampa, Florida US revealed that atmospheric deposition was the major contributor of NO_3–N in residential urban catchments (35 to 71%), followed by chemical fertilizers (1 to 49%), and soil and organic materials (7 to 33%) [26, 27].

While there are numerous studies investigating the sources of NO_3^- in urban waters, very few have investigated the sources of organic N forms (DON and PON). In a number of urban landscapes, particulate matter and organic detritus are considered main contributors to nutrient enrichment in stormwater runoff [11, 28, 29]. Bratt et al. [11] investigated stormwater outflow in a residential area in Minnesota, US and suggested that leaf litter was a potential source of N via transport of street PON during high rainfall events and also due to decomposition of litter by microbes. Janke et al. [28] in Minneapolis, US across 19 urban watersheds demonstrated that TN was strongly correlated to street trees canopy ($r=0.68$, $p<0.05$), suggesting that tree litter (e.g., leaves, seeds, flowers) contributes to N loading in stormwater. The study also showed that organic N concentration which was the primary N form (mean: 71% of TN across all sites) was strongly correlated with street canopy ($r= 0.71$, $p<0.001$). For source tracking of PON sources, dual isotopes of ^{13}C and ^{15}N have been reported as an effective tool [30-32].

The objectives of this study were to investigate (1) all N forms, including DON and PON, in urban runoff, (2) the influence of rainfall variables (e.g., total rainfall, intensity, duration, runoff volume, antecedent dry period) on inorganic and organic N loads to runoff, and (3) the sources of N in stormwater runoff, ~~to aid in developing management strategies for achieving TN reduction in stormwater runoff~~. Specifically, this study is one of the first to look at all N forms, including organic N, and include a source tracking component for PON.

Materials and methods

Site description

The study site was located in Lakewood Ranch, a residential neighborhood, in Manatee County, Florida, United States, which is a planned community with population of 29,411 in 2018

(67% increase over the population of 19,816 in 2010). Total land area in the community is 6,153 ha with 35 ha of water features that include 321 stormwater retention ponds. Surface runoff from residential areas enters into the stormwater ponds, which then flows to the Braden River, a sub-basin of the Manatee River and part of the Tampa Bay estuary watershed. Within the community, a study catchment of 3.89 hectares was identified, which consisted of 57.1% impervious surfaces (i.e. houses and roads) and 42.9% pervious surfaces (i.e. tree canopy and turfgrass). Surface runoff from this catchment enters into stormwater curbs and then into the stormwater pipe before draining into a stormwater pond of 0.64 ha (Fig.1 and S1 Table). Like other counties in Florida, Manatee county (our study site) is subjected to rainy season N fertilizer ban (Ordinance 11-20) adopted and enacted by the Board of County Commissioners of Manatee County, Florida. (http://www.mymanatee.or[illegible]epartments/parks_natural_resources/environmental_protection/the_manatee_county_fertilizer_ordinance/). This ban prohibits the application of N fertilizers during summer months from June 1 to September 30

Fig 1. Location map of the study site and residential catchment and various instruments installed in the stormwater outlet pipe including A) laser flow meter, B) rain gauge, and C) autosampler.

Study site instrumentation

Field instruments (laser flowmeter, rain gauge, and autosampler) were installed at the end of the stormwater pipe that delivers runoff from the catchment to the stormwater pond (Fig. 1B). A laser flowmeter (LaserFlow Non-contact Velocity Sensor) was used to measure flow and a rain

gauge was used to measure rainfall (Teledyne ISCO Inc., Nebraska, US). A refrigerated autosampler was used to collect runoff water samples (Avalanche, Teledyne ISCO Inc., Nebraska, US). Autosampler was triggered to collect runoff samples when the rain gauge recorded 2.54 mm of rainfall in 10 minutes and/or the minimum level of water depth in the pipe (detected by the flowmeter) was 19.05 mm for 5 minutes. This criteria allowed ~~to collect~~ runoff samples over the several storm events of various duration and magnitude. The autosampler contained 14 plastic bottles of 950 ml each and kept the samples stored at 4°C until collection for each sampling event. The flow meter was connected to a modem, which provided online connection for all data (allowed rainfall amount and duration, runoff volume, and sample collection times) download using the FlowLink 5.1 software (Teledyne ISCO Inc., Nebraska, US). The online connectivity system enabled ~~to monitor and manage~~ the sampling program ~~of the flowmeter~~ remotely, using the FlowLink 5.1 software.

Sample collection and preparation

Sampling was conducted in 2016 to capture the summer rainy season (June to September) and a month (May) preceding it. The plastic bottles from the autosampler were replaced ~~with the empty one~~ at the end of each storm event or when all 14 bottles were full, which were transported on ice to the laboratory within 24 h of collection. Prior to sampling, all bottles were acid washed (10% hydrochloric acid, HCl) and rinsed with deionized water.

Nitrogen concentrations analysis

A subsample of the stormwater samples (n=218) was vacuum-filtered using 0.45 µm glass fiber filters (GF/F) (Pall Corporation, Ann Arbor, MI) within 24 h of collection and analyzed using an auto analyzer (AA3, Seal Analytical Inc., Mequon, WI) for NH_4–N and NO_x–N (NO_3–N + NO_2–N) with USEPA Method 350.1 (USEPA, 1993) and 353.2 (US-EPA, 1993),

respectively. The filtered (0.45 μm) and unfiltered samples were analyzed for total dissolved N (TDN) and TN, respectively, using oxidative combustion-chemiluminescence by Total Organic Carbon Analyzer (TOC-L CPH/CPN, Shimadzu Corp., Columbia, MD). Other N forms were calculated as follows: PON = TN – TDN; DON = TDN – (NO_x–N + NH_4–N).

Flow-weighted mean concentrations (FWMC) in mg L^{-1} were used to quantify the weighted concentration proportional to corresponding flow volume. The FWMC for each parameter are derived from the concentrations and flow volume for each sample during a specified window of time. This equation allows the concentration in each sample to be considered in light of the time and associated flow volume [33, 34]. The equation is as follows:

$$FWMC = \sum_{1}^{n} \frac{(ci * ti * qi)}{(ti * qi)} \qquad (2\text{-}1)$$

where ci = concentration in the ith sample

ti = time (min) window of the ith sample

qi = flow volume in the ith sample

Water isotopes analysis

Subsamples of filtered stormwater were refrigerated at 4ºC for stable isotopes of water (H_2O) i.e. oxygen ($\delta^{18}O$–H_2O) and hydrogen (δD–H_2O). A total of 10 rainfall samples from 10 sampling events and 176 runoff samples (additional 14 samples from event #1 and 7 samples from event #2 were omitted due to technical error) were analyzed for the isotopic composition of H_2O using Chlortetracycline Liquid Chromatography Prep and Load (CTC LC-PAL) autosampler that was coupled with an off-axis integrated cavity output spectroscopy (OA-ICOS) water isotope analyzer (LWIA, Los Gatos Research, Mountain View, CA). A detailed description of H_2O stable isotope analysis can be found in Lis et al. [35]. All stable isotopes

values were reported as per mil (‰) according to Vienna Standard Mean Ocean Water (VSMOW) standards for O, and deuterium (D) with δ (‰) = 1000 x [(R_{sample}/$R_{standard}$)] – 1, where *R* represents the measured isotopic ratios of $^{18}O/^{16}O$, and D/H, respectively.

Nitrate isotopes analysis

The filtered stormwater samples were frozen for NO_3^- isotopic analysis i.e. $\delta^{18}O–NO_3^-$ and $\delta^{15}N–NO_3^-$. Out of 22 storm events, runoff samples from 12 events (n=176), and 12 rainfall samples were analyzed for the isotopic composition of NO_3^-. The 176 runoff samples for the isotopic composition of NO_3^- were chosen based on the storm events that had more than 5 samples. Out of the 176 samples, 148 samples were suitable for NO_3^- isotopic analysis as the rest of the samples had NO_3^- concentrations below detection limit. Analysis of NO_3^- isotopes was conducted using the $AgNO_3$ method as described by [36]. All stable isotopes values are reported as per mil (‰) according to VSMOW standards for O and N with δ (‰) = 1000 x [(R_{sample}/$R_{standard}$)] – 1, where *R* represents the measured isotopic ratios of $^{18}O/^{16}O$, and $^{15}N/^{14}N$, respectively.

A Bayesian stable isotope mixing model Stable Isotope Analysis in R (MixSIAR) was used to quantify the contribution of NO_3^- sources as described elsewhere [27, 37]. Measured stable isotope data of our samples was compared with end member values of NO_3^- sources ($\delta^{18}O–NO_3^-$ and $\delta^{15}N–NO_3^-$) obtained from the literature [27, 38-40] to infer the NO_3^- sources. Potential end members considered here included atmospheric deposition, NH_4^+ fertilizer, NO_3^- fertilizer, nitrification, and soil and organic N in stormwater runoff (S2 Table). In the Bayesian mixing model, measured $\delta^{18}O–NO_3^-$ and $\delta^{15}N–NO_3^-$ values for each of the runoff samples from May to September 2016 (n=148) were assigned as "customer" and the mean isotopic values of the NO_3^- sources from the literature were assigned as "sources".

Particulate organic nitrogen sources analysis

Isotopic characterization of ^{13}C and ^{15}N in stormwater particulates was used to investigate the potential sources of PON, based on methods similar to Kendall et al. [41]. Stormwater runoff samples were filtered through weighted 0.45 µm filters (GF/F). The retained particulates on the filter paper were oven-dried at ~80 ºC. Potential sources of organic N from the residential catchment i.e. grass clippings, oak leaves, and acorns were collected in May 2016. These were washed with deionized water and oven-dried at ~80 ºC before grinding to a fine powder. Both filter paper and the powder were then analyzed for ^{13}C and ^{15}N, total carbon (C), and TN with an elemental analyzer (Costech ECS 4010 Elemental Combustion System) coupled to a mass spectrometer (Thermo Finnigan DeltaPlus XL, San Jose, California). All stable isotopes values are reported as per mil (‰) according to Vienna Pee Dee Belemnite (VPDB) standards for C, and atmospheric N_2 for N with δ (‰) = 1000 x $[(R_{\text{sample}}/R_{\text{standard}})] - 1$, where R represents the measured isotopic ratios of $^{13}C/^{12}C$ and $^{15}N/^{14}N$, respectively.

The isotopic mixing model, IsoError, as described by Phillips et al. [42] was used to investigate the contribution of each potential source to runoff PON. The IsoError model is freely available from the US Environmental Protection Agency (www.epa.gov/eco-research/stable-isotope-mixing-models-estimating-source-proportions). The potential sources/end members ^{13}C and ^{15}N values are presented in S3 Table.

Statistical Analysis

The SAS JMP Pro 13 software was used for statistical analysis in this study. Differences in the mean for response variables (i.e. TN, DON, PON, NH_4–N, NO_x–N, isotopes $\delta^{15}N$–NO_3^- and $\delta^{18}O$–NO_3^-) and rainfall variables (i.e. antecedent dry period, amount, intensity and duration of

rainfall), were input into a Pearson correlation to test for relationships among the variables. An alpha value equal to 0.05 was used as a threshold for statistical significance.

Results and discussion

Relationship between rainfall and stormwater runoff

Total rainfall during the study period (May to September 2016) was 105.5 cm (monthly mean: 19.4±3.9 cm), with the highest amount in August (26.2 cm) and the lowest in June (17.4 cm). Ten-year records (2006–2015) obtained from the Florida Automated Weather Network (FAWN) indicate that total rainfall ranged from 52 cm to 115 cm, with a mean value of 82±20 cm from May to September (Supporting information S1 Fig).

From May to September 2016, 22 out of 75 storm events met the threshold requirement of sampling (2.54 mm of rainfall for 10 minutes and/or minimum level of 19.05 mm for 5 minutes the in the pipe). The rainfall associated with the 22 events was 37.5 cm or 36% of total rainfall throughout the study period (105.5 cm), which included many small storms (<2.54 mm rain in 5 minutes) that did not meet the sampling threshold. The rainfall intensity associated with 22 storm events ranged from 0.2 to 5.1 cm hr^{-1} (Fig. 2). Total amount of stormwater runoff from the residential catchment during study period was 5.54 Million L ha^{-1}, whereas the runoff associated with 22 storm events was 1.68 million L ha^{-1} or 30% of total runoff.

Fig 2. Rainfall amount and intensity and stormwater flow associated with storm events from May to September 2016. The red triangles represent 22 sampled storm events.

As expected, rainfall and runoff were significantly correlated (r=0.89, p<0.001). The percent runoff was positively correlated with rainfall amount (r=0.71, p<0.001), duration (r=0.73, p<0.001), and intensity (r=0.44, p<0.05) meaning that more runoff was generated by higher intensity and longer duration of storm events. Fig 3A shows total rainfall, runoff during 75 storm events, including runoff associated with 22 sampled storm events. The frequency distribution shows that 22 storm events represented samples captured over runoff ranging from 10 to 80% (Fig 3B). The amount of rainfall was positively correlated with amount of runoff over 75 events, with slope of 0.68, suggesting that 68% of total rainfall was converted to runoff (Fig 3C). Similar findings regarding large rainfall events and stormwater runoff were reported by Brezonik and Stadelmann [43].

Fig 3. (A) Total rainfall and runoff, (B) frequency distribution of runoff, and (C) relationship between rainfall and runoff amount for total 75 storm events and sampled 22 storm events from May to September 2016.

Concentrations and loads of nitrogen forms in stormwater runoff

Overview of nitrogen forms

Concentrations of TN in 22 storm events ranged from 0.28 to 10.11 mg L^{-1}, with FWMC of 2.45±0.2 mg L^{-1} (S2 Fig). Among N forms, DON and PON had the highest concentrations in runoff samples. The FWMC of DON over 22 events ranged from 0.1 to 9.2 mg L^{-1} (grand mean: 1.2 mg L^{-1}), whereas PON ranged from 0.07 to 8.3 mg L^{-1} (grand mean= 0.9 mg L^{-1}).

Over the study period, the stormwater runoff transported 3.9 kg ha^{-1} of TN, with DON as the dominant contributor at 72% (2.8 kg ha^{-1}), followed by PON and NH_4–N at 10% (0.39 kg ha^{-1}), and NO_x–N at 8% (0.31 kg ha^{-1}) (Fig. 4A). Organic N forms (DON + PON) accounted for an average of 82% (range: 3 to 95%) of TN loads over the study period (Fig. 4B). Our results are similar to other studies that showed ON as the most dominant forms in stormwater samples [7, 10, 28].

Fig 4. (A) Loads of various N forms separated into four groups for easy visualization and (B) Percentage of N forms in 22 storm events from May to September 2016.

Inorganic nitrogen forms

Dissolved inorganic N (DIN) forms had lowest concentration in runoff samples with FWMC NO_x–N ranging from 0.02 to 0.6 mg L^{-1} (grand mean: 0.2 mg L^{-1}) and NH_4–N ranging from 0.1 to 0.5 mg L^{-1} (grand mean: 0.2 mg L^{-1}) (S4 Table). The relative contribution of DIN was greater at the beginning of the wet season, especially during storm events with high rainfall and runoff volume (Fig 3A and 4B). In this study, inorganic N load was significantly positive correlated with duration of rainfall (r= 0.46, p<0.05). The prolonged high runoff in events #1, #8 and #13 resulted in higher DIN loads as compared to other shorter duration storms (Fig. 4B).

Inorganic N was significantly correlated (S5 Table) with total rainfall (r= 0.90, p<0.001), and antecedent dry period (r= 0.94, p<0.001), suggesting that longer dry period followed by high rainfall events, such as the first event of the season, resulted in increased DIN contribution to the runoff. Li et al. [44] showed that first flush effects in an urban catchment in China were driven by antecedent periods and rainfall amount. Lewis and Grimm [22] observed greater

concentration of NH_4–N in their urban catchment due to the longer antecedent dry period. Dry season or antecedent dry period provide an extended window for accumulation of dry deposits and nutrient buildup on urban surface such as the streets and rooftops before the onset of summer storms [45, 46]. When storm events occur, these accumulated pollutants are washed off into stormwater runoff [47]. Kojima et al. [45] concluded that surface deposits i.e. road dust were the dominant contributor of NO_x–N in their urban catchment located in Chiba City, Japan.

In 22 storm events, concentration of all N forms decreased after the onset of rainfall as stormwater runoff volume increased (S2 Fig). Further, when rainfall decreased and eventually stopped, runoff volume decreased with time and TN concentrations slightly increased suggesting that the decrease in N forms in storm events was due to the dilution from rainfall. This trend is similar to study done by Miguntanna et al. [47] who also observed decrease in TN concentration with increase in rainfall duration.

Organic nitrogen forms

The composition of DON over 22 storm events was relatively consistent as DON was the most dominant N form during the study period (S4 Table). Among rainfall variables, DON load was significantly correlated only with intensity (r= 0.50, p<0.05), indicating that other variables such as rainfall amount and runoff amount did not significantly influence the relatively high contribution of DON to stormwater. This suggests that a significant amount of DON can be transported both at low and high rainfall events. In previous studies, DON sources were linked to organic fertilizers, soil organic matter, atmospheric deposition, and degradation of plant debris and leaf litters from urban landscapes [3, 9]. Hagedorn et al. [48] showed that there was increase in DON export in the summer as a result of high decomposer activity and availability of fresh leaf litter. Decomposition of leaf litter has been reported to be one of the main contributors to

DON in urban runoff [11, 49] suggesting that in order to reduce DON input in stormwater runoff, a control measure (e.g., street cleaning) prior to storm events could be used to eliminate the potential of PON decomposition to DON [29]. Selbig [29] showed that street cleaning and removal of leaf litter from street surfaces reduced the total N by 74% and total dissolved N by 71% as this reduced the potential of N leaching from accumulated particulates. They further suggested that coherence, constancy, and timing of street cleaning and leaf removal are important factors to be measured in constructing effective stormwater management practices. Hochmuth et al. [50] reported that stormwater has the potential to leach nutrients from plant debris instantly, thus removal of leaf litter and plant debris must be done as soon as possible prior to storm events. Furthermore, a number of researchers, including our previous work in Tampa Bay, have concluded that a portion of DON may be bioavailable and thus can be a source of water quality impairment in urban ecosystems [3, 8, 37, 51].

In urban landscapes, engineered headwaters flowing over impervious surfaces and storm gutters deliver dissolved organic matter and N during storm events rapidly and increase particulate inputs into urban stream networks [52]. Research has demonstrated that organic detritus and particulates can be the main contributors of N input into urban stormwater [29]. PON load was correlated with antecedent dry days (r= 0.67, p<0.001), rainfall amount (r= 0.73 p<0.001), and duration of rainfall (r= 0.55 p<0.001) (S5 Table) indicating more particulate accumulation during longer dry periods, and high rainfall and prolonged storm events transport more PON. Within our study catchment, we observed particulates such as plant debris and leaf litter trapped on the grates of storm gutters. Our runoff samples recorded unusual high PON (events #6 and #7) concentrations (>50% of TN) that led to high TN concentrations (S4 Table and Fig. 4). The data showed that these events had low rain and runoff volume, and occurred

after storms with low rain and runoff volume (<40% runoff) (Fig. 3A). Wei et al. [53] suggested that particulate matter might be intercepted by course surfaces during low stormwater runoff from previous events, thus debris stuck in the sampling drain was not flushed out resulting in high PON concentrations in the subsequent samples.

Given the contributions of DON and PON to stormwater in our study and the potential for DON to contribute bioavailable N in urban waters, we support efforts to incorporate organic N into N loading budgets and in designs for more effective stormwater management to improve the quality of urban waters as also suggested by other researchers [9, 54].

Variation in water isotopes in rainfall and stormwater runoff

In urban residential areas, stormwater runoff can originate from multiple water sources such as rainfall, municipal water used for irrigation, reclaimed water, or wastewater leaks. Therefore, we used water isotopes to investigate the origin of water in stormwater runoff samples. Stable isotopes of $\delta^{18}O–H_2O$ and $\delta D–H_2O$ are known as environmental isotopic tracers that allow inference of the hydrological processes and origin of water in the aquatic systems [55]. The values of $\delta^{18}O–H_2O$ and $\delta D–H_2O$ in our rainfall samples (n=10) ranged from –6.1‰ to –0.4‰ (mean: –3.5‰) and –36.4‰ to 10.1‰ (mean: –16.1‰), respectively (S3 Fig). The isotope values in rainfall are reported to be an indicator of weather conditions where lower $\delta^{18}O–H_2O$ and $\delta D–H_2O$ indicates higher precipitation amounts [56]. This observation was confirmed in our three high rainfall events (#1, #13 and #19) that had lower $\delta^{18}O–H_2O$ and $\delta D–H_2O$ values as compared to other events (S4 Fig).

The runoff samples (n=176) had $\delta^{18}O–H_2O$ ranging from –6.42‰ to 1.63‰ (mean: –3.15‰) and $\delta D–H_2O$ ranging from –43.35‰ to 18.64‰ (mean: –14.27‰). Our isotopic data is similar to a study conducted in South Florida during the summer wet season with mean values of $\delta^{18}O$–

H_2O at –3.38‰ and δD–H_2O at –16.5‰ [56]. The global meteoric water line (GMWL) is an equation interpreted as δD–H_2O = $8\delta^{18}O$–H_2O + 10, which represents the relationship between H and O isotopes of water [57]. The isotopic composition of our runoff samples was identically with GMWL as shown in SI Fig. S3 with δD–H_2O = $7.9\delta^{18}O$–H_2O + 11. The isotopic composition of runoff and rainfall samples at the study site was identical with each other with δD–H_2O = $8\delta^{18}O$–H_2O + 11, indicating that all runoff samples from 22 storm events originated from local rainfall and not from other sources (e.g., municipal water, reclaimed water, wastewater).

Our water isotopes data showed differences in rainfall and runoff samples, and also variation in individual events for both $\delta^{18}O$–H_2O and δD–H_2O (S4 Fig). These differences and variations might be attributed by the effects of rainfall water being transmitted by canopy such as throughfall and stemflow before falling into the ground and emerging as surface runoff [58] and also as condensation and evaporation processes that occurred as water was conveyed from land to stormwater network [27, 40].

Variation in nitrate isotopes in rainfall and stormwater runoff

The $\delta^{15}N$–NO_3^- and $\delta^{18}O$–NO_3^- in rainfall samples over 12 storm events (n=148) ranged from –4.43‰ to 5.69‰ (mean: –5.30‰) and 36.70‰ to 67.08‰ (mean: 60.52‰), respectively (S5 Fig). The rainfall $\delta^{15}N$–NO_3^- values were between the values reported by Felix et al. [38] and Buda and Dewalle [59], who reported $\delta^{15}N$–NO_3^- values of –5.7‰ to 11.3‰ (mean: 3.2‰) and –0.6‰ to 5.0‰ (mean: –2.9‰), respectively. The $\delta^{18}O$–NO_3^- were also similar to Felix et al (2015) and Buda and DeWalle [59] who reported values of 32.2‰ to 68.7‰ (mean: 47.9‰) and 11.9‰ to 70‰ (mean: 43.8‰), respectively. Studies on seasonal patterns showed that the isotope values of $\delta^{15}N$–NO_3^- and $\delta^{18}O$–NO_3^- in precipitation varied due to differences in NO_3^-

sources and atmospheric oxidation pathways [38, 40, 59]. For example, Hastings et al. [60] reported that $\delta^{15}N–NO_3^-$ was lower in cool season (October to March) compared to warm season (April to September) in their studied region (Bermuda) due to more lightning during the warm season. Studies demonstrated that in summer season, atmospheric reactions are dominated by oxidation of NO_x through hydroxyl radicals (OH) which causes lower $\delta^{18}O–NO_3^-$, whereas in winter season, reaction occur between NO_x and O_3 resulting in higher $\delta^{18}O–NO_3$ [59, 60]. The $\delta^{15}N–NO_3^-$ of stormwater runoff samples ranged from –9.72‰ to 8.06‰ (mean: 1.02‰), whereas $\delta^{18}O–NO_3^-$ of stormwater runoff samples ranged from –9.19‰ to 59.70‰ (mean: 26.93‰) (S5 Fig). The $\delta^{15}N–NO_3^-$ in our runoff samples showed variation within the individual storm events. The differences in the intra-storm variation of $\delta^{15}N–NO_3^-$ ranged from 0.39‰ to 10.06‰ for all 12 storm events (mean: 5.04‰). The maximum variation in $\delta^{15}N–NO_3^-$ was in event (#19) with 10.06‰.

The $\delta^{18}O–NO_3^-$ in our runoff samples also showed variation within individual storm events (S5B Fig). The intra-storm variation of $\delta^{18}O–NO_3^-$ ranged from 14.17 to 56.67‰ for all 12 storm events (mean: 33.28‰). The maximum difference in isotope $\delta^{15}N–NO_3^-$ was in event #8 with 56.67‰. These results showed that the variation in the $\delta^{15}N–NO_3^-$ and $\delta^{18}O–NO_3^-$ in our stormwater runoff samples might be attributed to the changes in sources of atmospheric NO_3^- and mixing of NO_3^- from different sources in the catchment during storm events [26, 59, 61, 62].

Changing sources of nitrate in storm events

The isotopic values of $\delta^{15}N–NO_3^-$ and $\delta^{18}O–NO_3^-$ in our samples were in range observed for multiple sources such as atmospheric deposition (that includes vehicle emission and lightning), nitrification, inorganic fertilizer (NO_3^- and NH_4^+), and soil and organic N (S2 Table and Fig 5). The $\delta^{18}O–NO_3^-$ from –10‰ to 10‰ has been suggested to be indicative of nitrification, which

can be calculated using this formula: $\delta^{18}O–NO_3^-$ = 1/3 $\delta^{18}O–O_2$ + 2/3 $\delta^{18}O–H_2O$ [40]. The expected values of $\delta^{18}O–NO_3^-$ for nitrification in our samples, obtained from the equation, ranged from 3.55‰ to 8.92‰. Further, we observed shifting pattern of NO_3^- sources in individuals storm events especially in events with high and longer duration of rainfall such as events 1, 8, 13, and 19 (Fig 6). In these four events, atmospheric deposition is initially the main NO_3^- source, however, as the storm events progressed, the main source of NO_3^- changed to NO_3^- fertilizer, eventually including multiple other sources such as NH_4^+ fertilizers, nitrification, and soil and organic N. This pattern suggests that when the storm events started, the earliest runoff samples were from the direct atmospheric deposition. As the storm event progressed, the runoff began carrying other landscape sources of NO_3^-. This finding may also explain the higher composition of inorganic N in stormwater events with high and prolonged storm events, as rainfall become enriched with other N sources from the catchment. This is the first direct evidence that stormwater runoff can mobilize and transport inorganic N fertilizers from urban landscapes and therefore there might be some validity to claim that urban N fertilizers have the potential to be carried in stormwater runoff to receiving water bodies.

Fig 5. Stable isotopes of $\delta^{15}N$-NO_3^- and $\delta^{18}O$-NO_3^- in 12 sampled storm events from May to September 2016. Boxes indicate the range of the $\delta^{15}N–NO_3^-$ and $\delta^{18}O–NO_3^-$ values for NO_3^- sources according to (A) Kendall et al. (2007), Heaton (1990), and Felix et al. (2015) as shown in S2 Table.

Fig 6. Stable isotopes of $\delta^{15}N–NO_3^-$ and $\delta^{18}O–NO_3^-$ in four individuals storm events (A) event 1 (n=14), (B) event 8 (n=27), (C) event 13 (n=39) and (D) event 19 (n=10) from May to September 2016.

Identifying nitrate sources in stormwater runoff

We used Bayesian mixed model to separate the contribution of various sources of NO_3^-. Consistent with our previous research in Tampa Bay region [26], we found that atmospheric deposition was an important contributor of NO_3^- in stormwater runoff (34.9%), followed by NO_3^- fertilizer (24.7%), NH_4^+ fertilizers (17.2%), nitrification (14.8%), and soil and organic N sources (8.4%) (Fig. 7A). In this study, we applied the Bayesian mixing model to individual storm events in order to identify the NO_3^- sources to capture source variability between storm events. Among 12 storm events, 7 events had isotopic signatures of NO_3^- dominated by atmospheric deposition (mean: 30.1 to 63.5%), 3 events were dominated by NO_3^- fertilizer (mean: 31.6 to 43.5%), 1 event (#7) was dominated by NH_4^+ fertilizers (48.1%), and another event (#13) was dominated by nitrification (28.3%) (Fig. 7B). The changing sources contribution in different events highlight variable nature of NO_3^- sources in the landscape.

Fig 7. (A) Mean percent contribution of five nitrate sources from May to September 2016 and (B) Mean percent of NO_3^- sources for selected storm events (n=12) from May to September 2016.

Overall, NO_3–N in our samples was dominantly contributed by atmospheric deposition (Fig. 7A), which occurs as both dry and wet deposition [24, 63]. The mean contribution of atmospheric deposition to NO_3^- in our study was 34.9% (range: 7.4 to 63.5%). These results are similar to studies by other researchers that showed atmospheric deposition was a dominant source of NO_3–N in their runoff samples, ranging from 42 to 71% [26], 43 to 50% [63], and up to 94% [64]. Baral et al. [65] demonstrated that the contribution of atmospheric N to stormwater

is particularly important for small storms by showing that atmospheric deposition was a dominant contributor of NO_3–N in runoff with rainfall events less than 32 mm. Our study confirms this finding as 83% of our rainfall events were from storms with <30 mm rainfall.

Sources of NO_3^- from lightning, vehicle emissions, and coal-fired power plants are also defined as from atmospheric deposition. Based on isotopic values of $\delta^{15}N$–NO_3^-, our samples showed lightning and vehicle emissions as potential contributors to NO_3^- in runoff (Fig 5). Previously reported $\delta^{15}N$–NO_3^- values are –0.5‰ to 1.4‰ for lightning and 3.4‰ to 17‰ for fossil fuel combustion emissions [38, 39]. Lightning has been reported to be a source of NO_3^- in rainfall [59, 60]. Studies have shown that dry deposition of N from automobiles emissions can be a significant N source in urban stormwater runoff through atmospheric deposition [38, 59]. Our study location was in Bradenton, FL, an urban catchment with annual average daily traffic between 15,000 and 130,000 automobiles, suggesting that the high number of automobiles can be one of the contributors to N in the urban catchment. The isotopic composition of our samples were also in line with reported values for NO_3^- from coal-fired power plants reported by Heaton [66] and Felix et al. [38] as $\delta^{15}N$–NO_3^- ranged from 4.8‰ to 13‰. There are three power plant facilities within 40 km of the study site suggesting NO_x emission from coal-fired power plant as a potential source of atmospheric NO_3^- in our catchment. To our knowledge, there are no reported value of $\delta^{18}O$–NO_3^- for vehicle emissions, coal-fired power plants, and lightning, thus, the quantitative analysis on the relative contribution of these sources to NO_3^- in our catchment could not be conducted.

Inorganic N fertilizers (NO_3^- and NH_4^+) were the 2nd and 3rd largest contributors to NO_3–N in our runoff samples (Fig. 7A). The Bayesian mixing model indicated that NO_3^- fertilizer contributed 13.5% to 43.5% (mean: 24.7%), whereas NH_4^+ fertilizer contribution to NO_3–N was

4.6% to 48.1% (mean: 17.2%). Several studies on potential sources of anthropogenic N found that N input from fertilizer was the largest source of anthropogenic N fluxes from landscapes into the aquatic ecosystems [67-69]. Inorganic N fertilizers are commonly used in residential areas as part of landscape management practices to maintain plant quality. Inorganic N fertilizer was the dominant contributor in events 7, 15 and 19, which were storm events with high rainfall amount that resulted in high runoff flows. Even though our study was conducted during the summer season when application of N fertilizers is prohibited in this residential catchment from June 1 to September 30 (exception of event 1), the source of inorganic fertilizers might be from the residues of long-term controlled release fertilizers applied before the ban period (i.e. June 1). The premise behind the ban is that high rainfall causes more stormwater runoff that can carry inorganic N fertilizer into the surface waters [70].

The mean contribution of nitrification to NO_3^- was 14.8% (range: 4.9 to 28.3%) in our runoff samples suggesting that the runoff water carried a part of NH_4–N that was nitrified, contributing to NO_3–N. Nitrification was the main contributor of NO_3-N in event #13 (Fig. 7B). Among 12 storm events, event 13 was the longest storm event recorded with high rainfall amount suggesting that more NO_3–N from *in situ* soil nitrification was leached out and carried by stormwater runoff during this storm event. Soil and organic N was the lowest contributor to NO_3–N in the study (Fig 7A). The mixing model suggested that soil and organic N contributed about 3.1 to 15.4% of NO_3–N (mean: 8.4%). Numerous studies reported that organic N originating from household wastewater was the main source of NO_3–N in surface and ground waters [71, 72], however, this is not the case in our residential catchment, as all wastewater is connected and delivered to a wastewater treatment plant. Further, our water isotope results indicated that stormwater runoff throughout the study period originated from local rainfall and

not from wastewater or other water sources. This finding is similar to our stormwater runoff studies conducted in other Florida residential catchments [26, 27]. It is noteworthy to mention that this study and our other Florida studies were conducted in relatively newer residential areas (<20 years old) and hence these sites have newer wastewater and stormwater infrastructure that is less prone to leakage from wastewater as compared to other cities in the world. Lastly, the organic materials such as leaf-litter, grass clipping, animal waste, and soil organic matter are the possible sources of 8.4% of NO_3^- in our residential catchment [27, 67].

Sources of particulate organic nitrogen in stormwater runoff

The values of $\delta^{15}N$ and $\delta^{13}C$ in stormwater runoff particulates i.e. PON (n=163 in 19 storm events) ranged from –1.99‰ to 6.27‰ (mean: –1.03±1.38‰) and –28.31‰ to –19.46‰ (mean: –23.04±1.73‰), respectively (Fig. 8). These isotopic values appeared to be from mixture of St. Augustine (*Stenotaphrum secundatum*) grass clippings, acorns, and leaves from live oak (*Quercus virginia*) trees. The mean ^{15}N for grass clippings, acorns, and oak leaves was –0.46 ‰ (range: –1.93 to 0.68), 1.58‰ (range: 1.55 to 1.60), and –1.24‰ (range: –1.70 to –0.83), respectively (Fig. 8). Whereas the mean ^{13}C for St. Augustine grass, acorns, and oak leaves was –14.2‰ (range: –17.8 to –11.6), –29.4‰, (range: –30.79 to –28.44) and –28.8‰ (range: –29.90 to –27.41), respectively. This data were modelled using IsoError mixing model (Phillips et al., 2005), which showed that acorns (0.41 or 41%), followed by grass clippings (0.32 or 32%), and oak leaves (0.27 or 27%) were the dominant contributor of PON in stormwater runoff (Fig. 8).

Fig 8. Values of $\delta^{15}N$ and $\delta^{13}C$ for endmembers (acorns, oak leaves, and St. Augustine grass) and runoff particulate organic N (PON) samples (blue crosses). Source proportions represent IsoError mixing model results and are estimates of the proportion of each source in the runoff samples.

The particulates from oak detritus (acorn and oak leaves) accounted for 59% of PON in our stormwater samples. Acorns drop from live oak trees in Florida during October each year, which then decompose on the ground for the following several months. Live oak trees naturally shed old leaves in spring (February to March) as the new leaves emerge (Ong, 2008), which then slowly decompose over the dry season. When the wet season begins in June, these partly decomposed materials (acorns and oak leaves) are carried by the stormwater runoff into the gutter and then contribute PON in runoff. This hypothesis is supported by several studies that demonstrated decomposition of leaf litter as a contributor to dissolved N in stormwater [11, 28, 29, 73]. These studies suggested particulates were decomposed by vehicles activity on the road surface, movement during storm events, and further decomposition in road gutters, thus contributing PON while DON was gained from leaching of freshly fallen litter.

Urbanization often leads to changes in vegetation from trees to grasses [64]. In our study catchment, turfgrass (St. Augustine) covered about 51% of total area (S1 Table), thus making grass clippings one of the most abundant source of PON in our catchment. Newcomer et al. [74] suggested that grass clippings are a potential source of labile N that can be readily mineralized. Lusk et al. [75] showed that DON was the main N form in leachate from turfgrass (St. Augustine) and suggested that root and microbial exudates in turf systems can convert inorganic fertilizer N to organic N that can be leached as DON in short periods of times (days to week).

Conclusions

This study was conducted to investigate the composition of N forms and sources of NO_3^- and PON in stormwater runoff. Among all N forms, DON was the dominant N form (mean: 47%) in stormwater runoff from May through September 2016 suggesting that management of runoff in terms of N should target not only inorganic N, but also organic N. Among rainfall variables, DON was positively correlated to only intensity, indicating that higher intensity of rain may be flushing out DON from soils and/or causing leaching of DON from particulates in the catchment. Statistical analysis showed that longer dry season and high rainfall amounts are more significant drivers for DIN transport to water bodies. Nitrate loading to stormwater runoff was derived from the mixing of multiple sources with atmospheric deposition as the dominant NO_3–N source (34.9%) followed by NO_3^- fertilizer (24.7%), NH_4^+ fertilizer (17.2%), nitrification (14.8%), and soil and organic N (8.4%). The isotopic data showed a shifting pattern of NO_3^- sources in events with high and longer duration of rainfall, suggesting that stormwater management to reduce N transport should include approaches that incorporate both rainfall and stormwater runoff in designs, such as rain gardens, roof gardens, and permeable pavements. These low-impact development methods will not only reduce the momentum and erosive power of the stormwater and provide more time for the water to infiltrate into the ground, but also help to filter N before the stormwater enters into connected urban waters. The sources of PON in stormwater runoff were acorns (41%), grass clippings (32%), and leaves from live oak trees (27%) present in the residential catchment. The decomposition of PON is a potential contributor to DON loading in urban runoff, suggesting an approach for N reduction should take place prior to storm events such as removal of organic materials (e.g., leaf litters, grass clipping, animal wastes) from urban pervious and impervious surfaces.

Acknowledgements

This project was funded by grants from Florida Department of Environmental Protection and Center for Landscape Conservation and Ecology of the University of Florida. We thank Drs. Kati Migliaccio, Andrew Koeser, and John Thomas for serving on Jariani Jani's PhD committee. The first author thanks Ministry of Higher Education, Malaysia for providing a PhD fellowship. We thank Stefan Kalev, former MS student, for his help and assistance in troubleshooting field instruments and collecting stormwater runoff samples. This project would not have been possible without tremendous support, cooperation, and advocacy from residents and homeowner association of Lakewood Ranch community, located in Manatee County, Florida.

References

1. Fields S. Global nitrogen: cycling out of control. Environmental Health Perspectives. 2004;112(10):A556.
2. Lu C, Bowman D, Rufty T, Shi W. Reactive Nitrogen in Turfgrass Systems: Relations to Soil Physical, Chemical, and Biological Properties. Journal of environmental quality. 2015;44(1):210-8.
3. Bronk D, See J, Bradley P, Killberg L. DON as a source of bioavailable nitrogen for phytoplankton. Biogeosciences. 2007;4(3):283-96.
4. Howarth RW, Marino R. Nitrogen as the limiting nutrient for eutrophication in coastal marine ecosystems: evolving views over three decades. Limnology and Oceanography. 2006;51(1):364-76.

5. Smith VH, Joye SB, Howarth RW. Eutrophication of freshwater and marine ecosystems. Limnology and Oceanography. 2006;51(1part2):351-5.

6. Seitzinger SP, Sanders R, Styles R. Bioavailability of DON from natural and anthropogenic sources to estuarine plankton. Limnology and Oceanography. 2002;47(2):353-66.

7. Li L, Davis AP. Urban stormwater runoff nitrogen composition and fate in bioretention systems. Environmental science & technology. 2014.

8. Lusk M, Toor G. Dissolved organic nitrogen in urban streams: Biodegradability and molecular composition studies. Water Res. 2016;96:225-35. doi: 10.1016/j.watres.2016.03.060. PubMed PMID: WOS:000376211700023.

9. Lusk M, Toor G. Biodegradability and Molecular Composition of Dissolved Organic Nitrogen in Urban Stormwater Runoff and Outflow Water from a Stormwater Retention Pond. Environmental Science & Technology. 2016;50(7):3391-8. doi: 10.1021/acs.est.5b05714. PubMed PMID: WOS:000373655800012.

10. Taylor GD, Fletcher TD, Wong TH, Breen PF, Duncan HP. Nitrogen composition in urban runoff—implications for stormwater management. Water Res. 2005;39(10):1982-9.

11. Bratt AR, Finlay JC, Hobbie SE, Janke BD, Worm AC, Kemmitt KL. Contribution of Leaf Litter to Nutrient Export during Winter Months in an Urban Residential Watershed. Environmental Science & Technology. 2017;51(6):3138-47. doi: 10.1021/acs.est.6b06299.

12. Kaushal SS, Groffman PM, Band LE, Elliott EM, Shields CA, Kendall C. Tracking Nonpoint Source Nitrogen Pollution in Human-Impacted Watersheds. Environmental Science & Technology. 2011;45(19):8225-32. doi: 10.1021/es200779e.

13. Yang Y-Y, Lusk MG. Nutrients in Urban Stormwater Runoff: Current State of the Science and Potential Mitigation Options. Current Pollution Reports. 2018;4(2):112-27. doi: 10.1007/s40726-018-0087-7.

14. Denault C, Millar RG, Lence BJ. ASSESSMENT OF POSSIBLE IMPACTS OF CLIMATE CHANGE IN AN URBAN CATCHMENT 1. JAWRA Journal of the American Water Resources Association. 2006;42(3):685-97.

15. Langeveld JG, Schilperoort RP, Weijers SR. Climate change and urban wastewater infrastructure: there is more to explore. Journal of hydrology. 2013;476:112-9.

16. Willems P. Revision of urban drainage design rules after assessment of climate change impacts on precipitation extremes at Uccle, Belgium. Journal of Hydrology. 2013;496:166-77.

17. Hathaway J, Tucker R, Spooner J, Hunt W. A traditional analysis of the first flush effect for nutrients in stormwater runoff from two small urban catchments. Water, Air, & Soil Pollution. 2012;223(9):5903-15.

18. Lee J, Bang K, Ketchum L, Choe J, Yu M. First flush analysis of urban storm runoff. Science of the Total Environment. 2002;293(1):163-75.

19. Schiff KC, Tiefenthaler LL. Seasonal Flushing of Pollutant Concentrations and Loads in Urban Stormwater1. Wiley Online Library; 2011.

20. Schiff K, Tiefenthaler L, Bay S, Greenstein D. Effects of rainfall intensity and duration on the first flush from parking lots. Water. 2016;8(8):320.

21. Liu H, Jeong J, Gray H, Smith S, Sedlak DL. Algal uptake of hydrophobic and hydrophilic dissolved organic nitrogen in effluent from biological nutrient removal municipal wastewater treatment systems. Environmental science & technology. 2011;46(2):713-21.

22. Lewis DB, Grimm NB. Hierarchical regulation of nitrogen export from urban catchments: interactions of storms and landscapes. Ecological Applications. 2007;17(8):2347-64.

23. Carey RO, Hochmuth GJ, Martinez CJ, Boyer TH, Nair VD, Dukes MD, et al. Regulatory and Resource Management Practices for Urban Watersheds: The Florida Experience. HorTechnology. 2012;1 of 4(4):418-29.

24. Divers MT, Elliott EM, Bain DJ. Quantification of Nitrate Sources to an Urban Stream Using Dual Nitrate Isotopes. Environmental Science & Technology. 2014;48(18):10580-7. doi: 10.1021/es404880j.

25. Hobbie SE, Finlay JC, Janke BD, Nidzgorski DA, Millet DB, Baker LA. Contrasting nitrogen and phosphorus budgets in urban watersheds and implications for managing urban water pollution. Proceedings of the National Academy of Sciences. 2017. doi: 10.1073/pnas.1618536114.

26. Yang Y-Y, Toor GS. δ15N and δ18O reveal the sources of nitrate-nitrogen in urban residential stormwater runoff. Environmental science & technology. 2016;50(6):2881-9.

27. Yang Y-Y, Toor GS. Sources and mechanisms of nitrate and orthophosphate transport in urban stormwater runoff from residential catchments. Water Res. 2017;112:176-84.

28. Janke BD, Finlay JC, Hobbie SE. Trees and Streets as Drivers of Urban Stormwater Nutrient Pollution. Environmental Science & Technology. 2017. doi: 10.1021/acs.est.7b02225.

29. Selbig W. Evaluation of leaf removal as a means to reduce nutrient concentrations and loads in urban stormwater. Science of the Total Environment. 2016;571:124-33. doi: 10.1016/j.scitotenv.2016.07.003. PubMed PMID: WOS:000383930400015.

30. Bardhan P, Naqvi SWA, Karapurkar SG, Shenoy DM, Kurian S, Naik H. Isotopic composition of nitrate and particulate organic matter in a pristine dam reservoir of western India: implications for biogeochemical processes. Biogeosciences. 2017;14(4):767-79.

31. Derrien M, Kim M-S, Ock G, Hong S, Cho J, Shin K-H, et al. Estimation of different source contributions to sediment organic matter in an agricultural-forested watershed using end member mixing analyses based on stable isotope ratios and fluorescence spectroscopy. Science of The Total Environment. 2018;618:569-78.

32. Gao X, Yang Y, Wang C. Geochemistry of organic carbon and nitrogen in surface sediments of coastal Bohai Bay inferred from their ratios and stable isotopic signatures. Marine pollution bulletin. 2012;64(6):1148-55.

33. Charbeneau RJ, Barrett ME. Evaluation of methods for estimating stormwater pollutant loads. Water Environment Research. 1998;70(7):1295-302.

34. Sansalone JJ, Buchberger SG. Partitioning and first flush of metals in urban roadway storm water. Journal of Environmental engineering. 1997;123(2):134-43.

35. Lis G, Wassenaar L, Hendry M. High-precision laser spectroscopy D/H and 18O/16O measurements of microliter natural water samples. Analytical chemistry. 2008;80(1):287-93.

36. Coplen T, Haiping Q, Révész K, Casciotti K, Hannon J. Determination of the δ (15 N/14N) and δ (18O/16O) of Nitrate in Water: RSIL Lab Code 2900. 2007.

37. Jani J, Toor GS. Composition, sources, and bioavailability of nitrogen in a longitudinal gradient from freshwater to estuarine waters. Water Res. 2018;137:344-54.

38. Felix JD, Elliott EM, Shaw SL. Nitrogen isotopic composition of coal-fired power plant NO x: influence of emission controls and implications for global emission inventories. Environmental Science & Technology. 2012;46(6):3528-35.

39. Hoering T. The isotopic composition of the ammonia and the nitrate ion in rain. Geochimica et Cosmochimica Acta. 1957;12(1-2):97-102.

40. Kendall C, Elliott EM, Wankel SD. Tracing anthropogenic inputs of nitrogen to ecosystems. Stable isotopes in ecology and environmental science. 2007;2:375-449.

41. Kendall C, Silva SR. Carbon and nitrogen isotopic compositions of particulate organic matter in four large river systems across the United States. Hydrological Processes. 2001;15:1301-46.

42. Phillips DL, Newsome SD, Gregg JW. Combining sources in stable isotope mixing models: alternative methods. Oecologia. 2005;144(4):520-7.

43. Brezonik PL, Stadelmann TH. Analysis and predictive models of stormwater runoff volumes, loads, and pollutant concentrations from watersheds in the Twin Cities metropolitan area, Minnesota, USA. Water Res. 2002;36(7):1743-57.

44. Li DY, Wan JQ, Ma YW, Wang Y, Huang MZ, Chen YM. Stormwater Runoff Pollutant Loading Distributions and Their Correlation with Rainfall and Catchment Characteristics in a Rapidly Industrialized City. Plos One. 2015;10(3):17. doi: 10.1371/journal.pone.0118776. PubMed PMID: WOS:000351183500042.

45. Kojima K, Murakami M, Yoshimizu C, Tayasu I, Nagata T, Furumai H. Evaluation of surface runoff and road dust as sources of nitrogen using nitrate isotopic compisition. Chemosphere. 2011;84:1716-22.

46. Tsai YI, Cheng MT. Characterization of chemical species in atmospheric aerosols in a metropolitan basin. Chemosphere. 2004;54(8):1171-81.

47. Miguntanna NP, Liu A, Egodawatta P, Goonetilleke A. Characterising nutrients wash-off for effective urban stormwater treatment design. Journal of Environmental Management. 2013;120(Supplement C):61-7. doi: https://doi.org/10.1016/j.jenvman.2013.02.027.

48. Hagedorn F, Bucher JB, Schleppi P. Contrasting dynamics of dissolved inorganic and organic nitrogen in soil and surface waters of forested catchments with Gleysols. Geoderma. 2001;100(1):173-92.

49. Duan S, Delaney-Newcomb K, Kaushal S, Findlay S, Belt K. Potential effects of leaf litter on water quality in urban watersheds. Biogeochemistry. 2014;121(1):61-80. doi: 10.1007/s10533-014-0016-9. PubMed PMID: WOS:000341908600004.

50. Hochmuth G, Nell T, Sartain J, Unruh JB, Martinez C, Trenholm L, et al. Urban water quality and fertilizer ordinances: Avoiding unintended consequences: A review of the scientific literature. 2012.

51. Osborne D, Podgorski D, Bronk D, Roberts Q, Sipler R, Austin D, et al. Molecular-level characterization of reactive and refractory dissolved natural organic nitrogen compounds by atmospheric pressure photoionization coupled to Fourier transform ion cyclotron resonance mass spectrometry. Rapid Communications in Mass Spectrometry. 2013;27(8):851-8. doi: 10.1002/rcm.6521. PubMed PMID: WOS:000316624400001.

52. Fork ML, Blaszczak JR, Delesantro JM, Heffernan JB. Engineered headwaters can act as sources of dissolved organic matter and nitrogen to urban stream networks. Limnology and Oceanography Letters. 2018;3(3):215-24.

53. Wei Z, Simin L, Fengbing T. Characterization of urban runoff pollution between dissolved and particulate phases. The Scientific World Journal. 2013;2013.

54. Van Kessel C, Clough T, van Groenigen JW. Dissolved organic nitrogen: an overlooked pathway of nitrogen loss from agricultural systems? Journal of environmental quality. 2009;38(2):393-401.

55. Kendall C, McDonnell JJ. Isotope tracers in catchment hydrology: Elsevier; 2012.

56. Price RM, Swart PK, Willoughby HE. Seasonal and spatial variation in the stable isotopic composition (δ18O and δD) of precipitation in south Florida. Journal of Hydrology. 2008;358(3-4):193-205.

57. Craig H. Isotopic variations in meteoric waters. Science. 1961;133(3465):1702-3.

58. Allen ST, Keim RF, Barnard HR, McDonnell JJ, Renée Brooks J. The role of stable isotopes in understanding rainfall interception processes: a review. Wiley Interdisciplinary Reviews: Water. 2017;4(1):e1187.

59. Buda AR, DeWalle DR. Using atmospheric chemistry and storm track information to explain the variation of nitrate stable isotopes in precipitation at a site in central Pennsylvania, USA. Atmospheric environment. 2009;43(29):4453-64.

60. Hastings MG, Sigman DM, Lipschultz F. Isotopic evidence for source changes of nitrate in rain at Bermuda. Journal of Geophysical Research: Atmospheres. 2003;108(D24).

61. Altieri K, Hastings M, Peters A, Sigman D. Molecular characterization of water soluble organic nitrogen in marine rainwater by ultra-high resolution electrospray ionization mass spectrometry. Atmospheric Chemistry and Physics. 2012;12(7):3557-71. doi: 10.5194/acp-12-3557-2012. PubMed PMID: WOS:000302806700025.

62. Hale RL, Turnbull L, Earl S, Grimm N, Riha K, Michalski G, et al. Sources and Transport of Nitrogen in Arid Urban Watersheds. Environmental Science & Technology. 2014;48(11):6211-9. doi: 10.1021/es501039t.

63. Anisfeld SC, Barnes RT, Altabet MA, Wu T. Isotopic Apportionment of Atmospheric and Sewage Nitrogen Sources in Two Connecticut Rivers. Environmental Science & Technology. 2007;41(18):6363-9. doi: 10.1021/es070469v.

64. Kaushal SS, Groffman PM, Band LE, Elliott EM, Shields CA, Kendall C. Tracking nonpoint source nitrogen pollution in human-impacted watersheds. Enviromental Science and Technology. 2011;45:8225-32.

65. Baral D, Fisher JR, Florek III MJ, Dvorak BI, Snow DD, Admiraal DM. Atmospheric contributions of nitrate to stormwater runoff from two urban watersheds. Journal of Environmental Engineering. 2017;144(2):05017009.

66. Heaton T. 15N/14N ratios of NOx from vehicle engines and coal-fired power stations. Tellus B. 1990;42(3):304-7.

67. Fissore C, Baker LA, Hobbie SE, King JY, McFadden JP, Nelson KC, et al. Carbon, nitrogen, and phosphorus fluxes in household ecosystems in the Minneapolis-Saint Paul, Minnesota, urban region. Ecological Applications. 2011;21(3):619-39. doi: 10.1890/10-0386.1. PubMed PMID: WOS:000290661300001.

68. Han Y, Fan Y, Yang P, Wang X, Wang Y, Tian J, et al. Net anthropogenic nitrogen inputs (NANI) index application in Mainland China. Geoderma. 2014;213:87-94.

69. Cao D, Cao W, Fang J, Cai L. Nitrogen and phosphorus losses from agricultural systems in China: a meta-analysis. Marine pollution bulletin. 2014;85(2):727-32.

70. Patil RH, Laegdsmand M, Olesen JE, Porter JR. Effect of soil warming and rainfall patterns on soil N cycling in Northern Europe. Agriculture, ecosystems & environment. 2010;139(1-2):195-205.

71. Carey RO, Hochmuth GJ, Martinez CJ, Boyer TH, Dukes MD, Toor GS, et al. Evaluating nutrient impacts in urban watersheds: Challenges and research opportunities. Environ Pollut. 2013;173:138-49. doi: 10.1016/j.envpol.2012.10.004. PubMed PMID: WOS:000313845500020.

72. De M, Toor GS. Fate of effluent-borne nitrogen in the mounded drainfield of an onsite wastewater treatment system. Vadose Zone Journal. 2015;14(12).

73. Hobbie S, Baker L, Buyarski C, Nidzgorski D, Finlay J. Decomposition of tree leaf litter on pavement: implications for urban water quality. Urban Ecosystems. 2014;17(2):369-85. doi: 10.1007/s11252-013-0329-9. PubMed PMID: WOS:000336382700001.

74. Newcomer TA, Kaushal SS, Mayer PM, Shields AR, Canuel EA, Groffman PM, et al. Influence of natural and novel organic carbon sources on denitrification in forest, degraded urban, and restored streams. Ecological Monographs. 2012;82(4):449-66.

75. Lusk MG, Toor GS, Inglett PW. Characterization of dissolved organic nitrogen in leachate from a newly established and fertilized turfgrass. Water Res. 2018;131:52-61.

Supporting information section

S1 Fig. (A) Annual and May to September rainfall from 2006 to 2016, (B) comparison of mean 10 years (2006-2015) and 2016 rainfall, and (C) comparison of monthly rainfall from May to September for 2006-2015 and 2016 in the study site located in Bradenton, Florida. Red bar in (A) indicates data of sampling year (2016).

S2 Fig. Flow-weighted mean concentrations of various N Forms in 22 sampled storm events from May to September 2016.

S3 Fig. Relationship between $\delta^{18}O–H_2O$ and $\delta D–H_2O$ for rainfall (n=10) and runoff samples (n=176) collected during 22 storm events from May to September 2016.

S4 Fig. Variation in stable isotope composition (A) $\delta^{18}O–H_2O$ and (B) $\delta D–H_2O$ in rainfall (n=10) and stormwater runoff samples (n=176) collected during 22 storm events from May to September 2016.

S5 Fig. Variation in stable isotope composition (A) $\delta^{15}N-NO_3^-$ and (B) $\delta^{18}O-NO_3^-$ in rainfall (n=12) and stormwater runoff samples collected during 22 storm events from May to September 2016.

S1 Table. Pervious and impervious area of residential catchment located in Lakewood Ranch, Bradenton, Florida, United States.

S2 Table. End member literature values of $\delta^{18}O–NO_3^–$ and $\delta^{15}N–NO_3^–$.

S3 Table. ^{13}C and ^{15}N of PON in various landscape sources and collected stormwater runoff samples.

S4 Table. Rainfall variables and flow-weighted mean concentration of N forms for 22 storm events from May to September 2016.

S5 Table. Pearson correlation among rainfall variables and N forms from May to September 2016.

Figures
Click here to download Figure Figures.docx

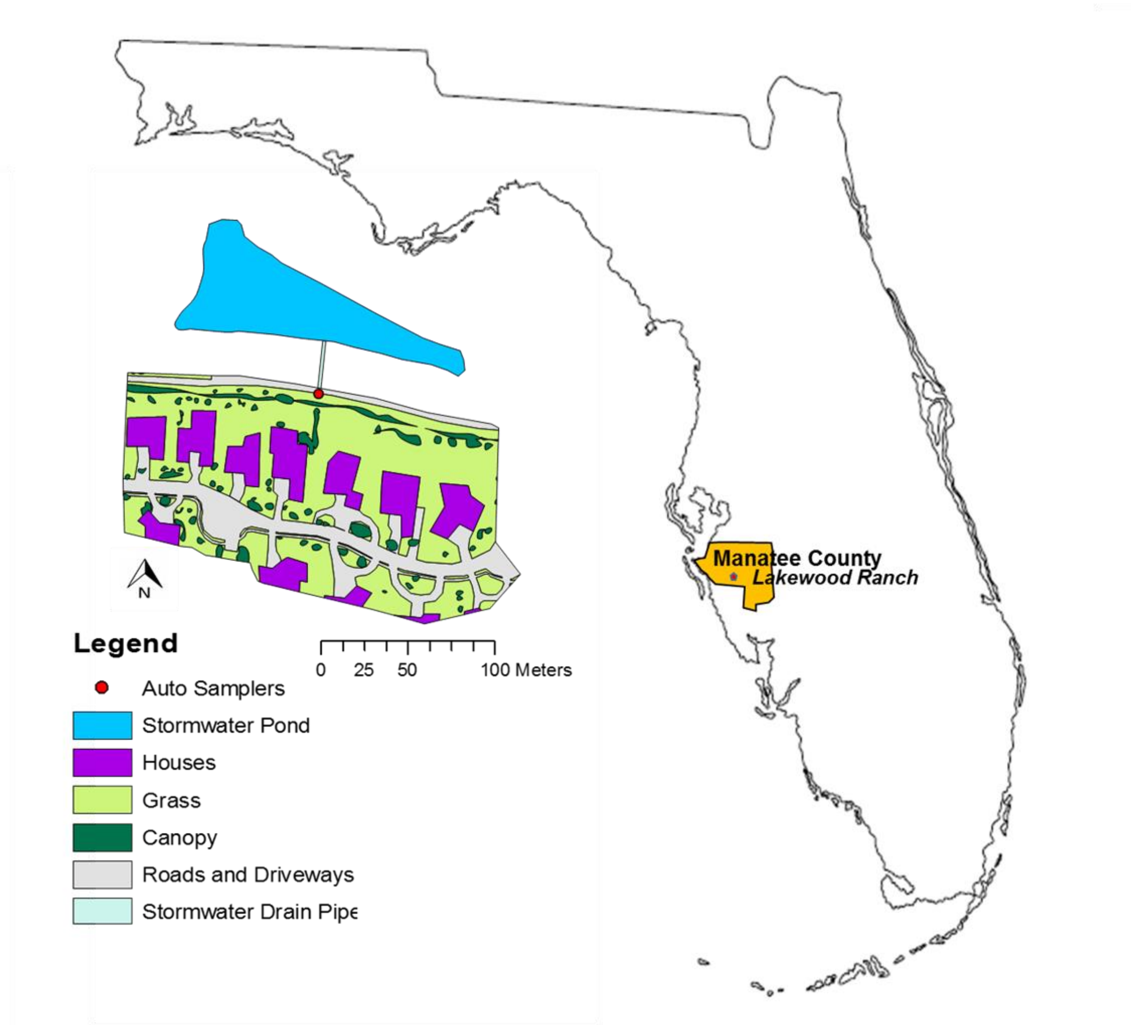

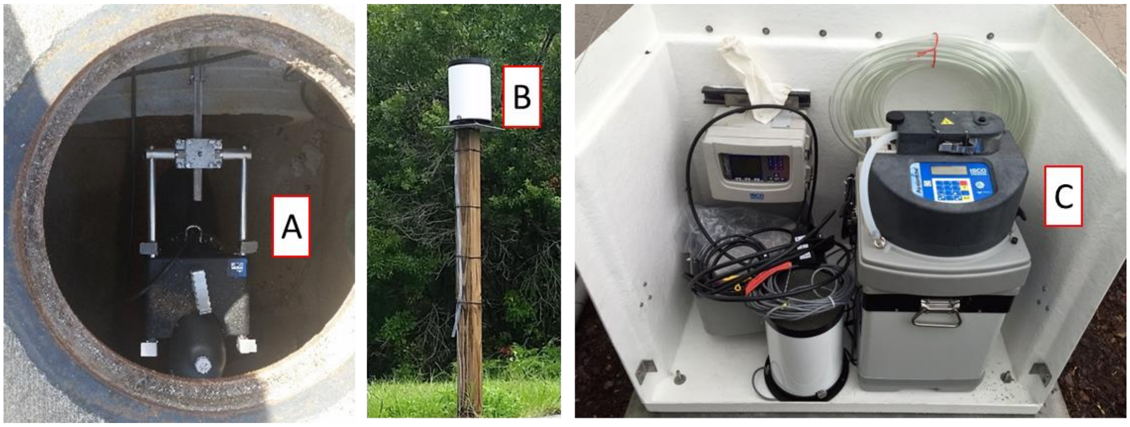

Fig 1. Location map of the study site and residential catchment and various instruments installed in the stormwater outlet pipe including A) laser flow meter, B) rain gauge, and C) autosampler.

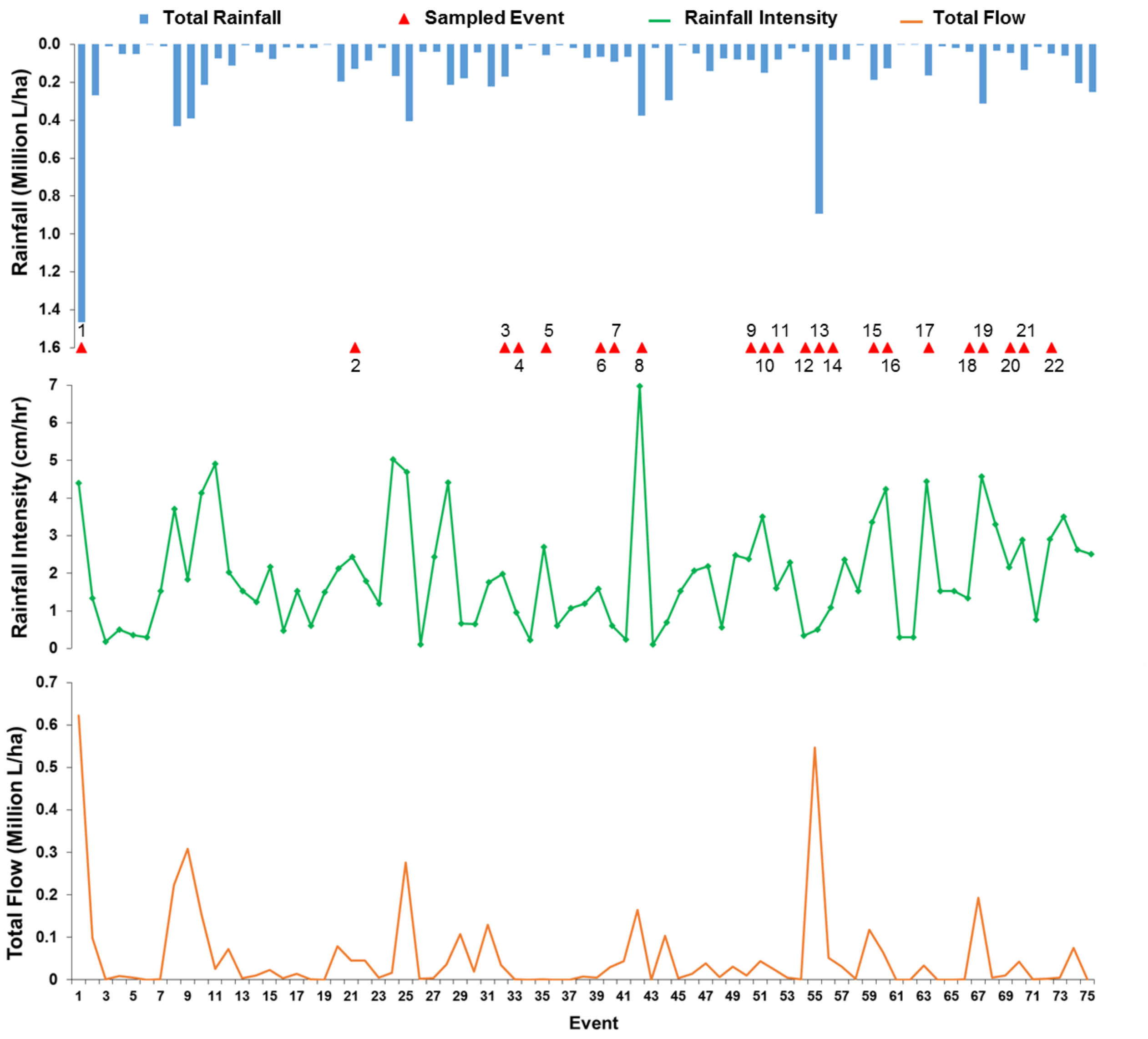

Fig 2. Rainfall amount and intensity and stormwater flow associated with storm events from May to September 2016. The red triangles represent 22 sampled storm events.

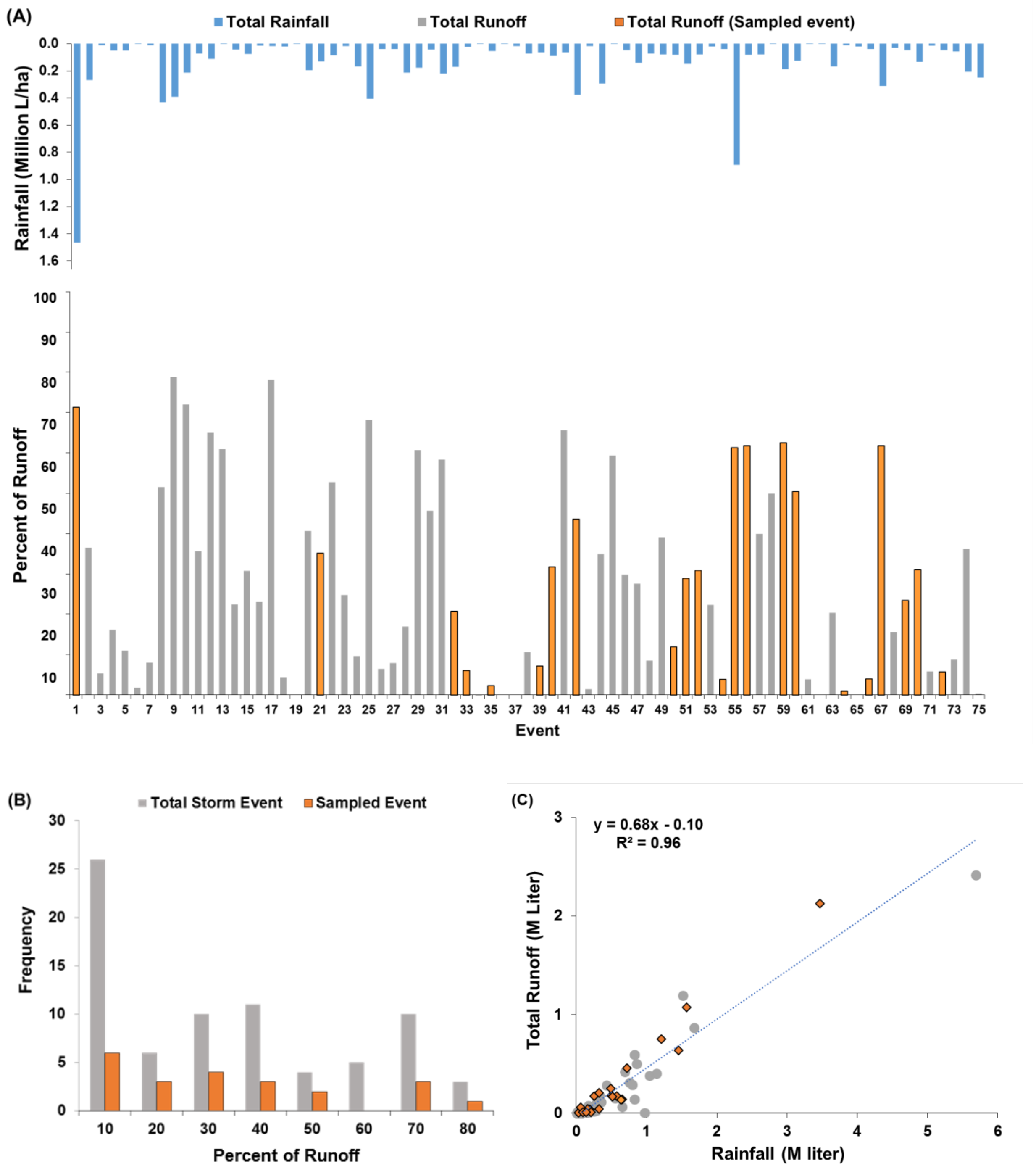

Fig 3. (A) Total rainfall and runoff, (B) frequency distribution of runoff, and (C) relationship between rainfall and runoff amount for total 75 storm events and sampled 22 storm events from May to September 2016.

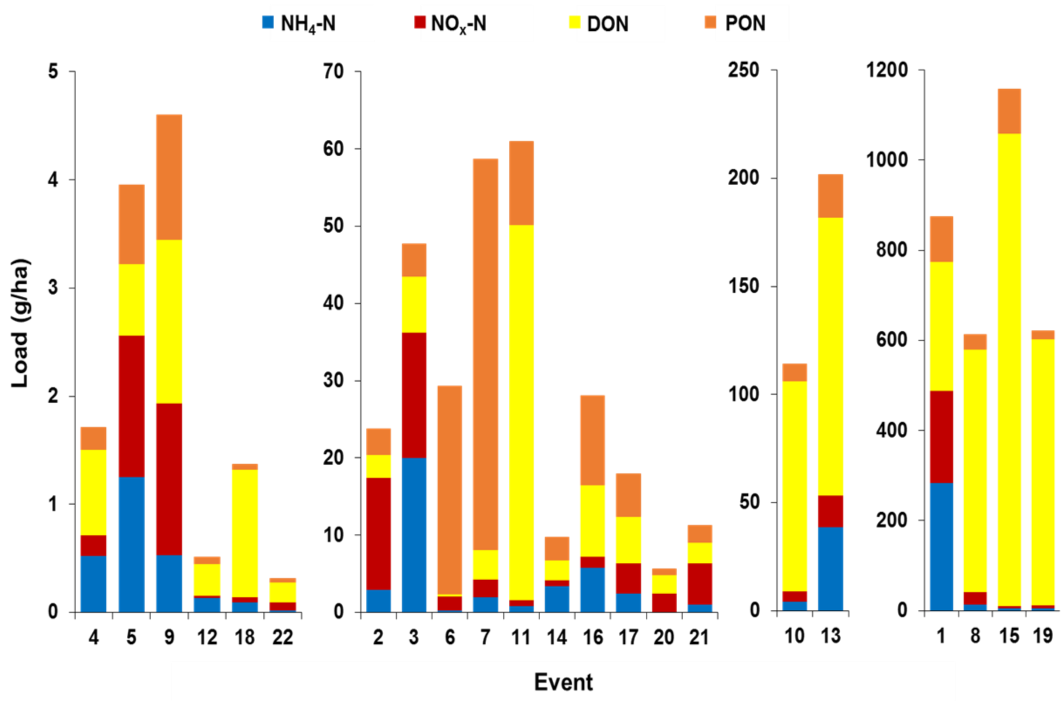

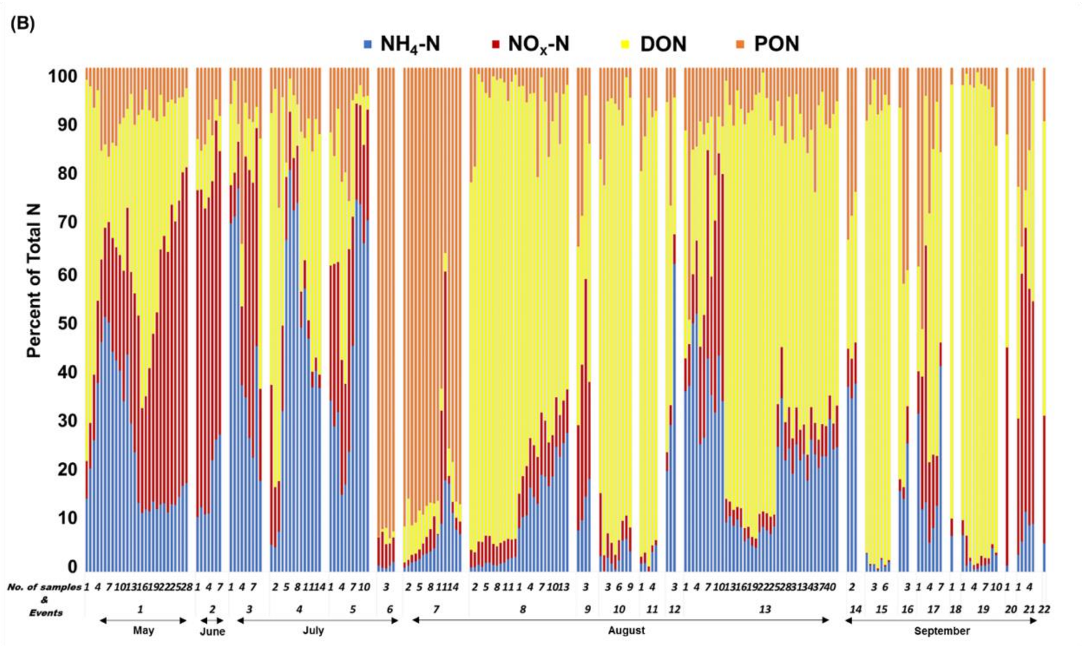

Fig 4. (A) Loads of various N forms separated into four groups for easy visualization and (B) Percentage of N forms in 22 storm events from May to September 2016

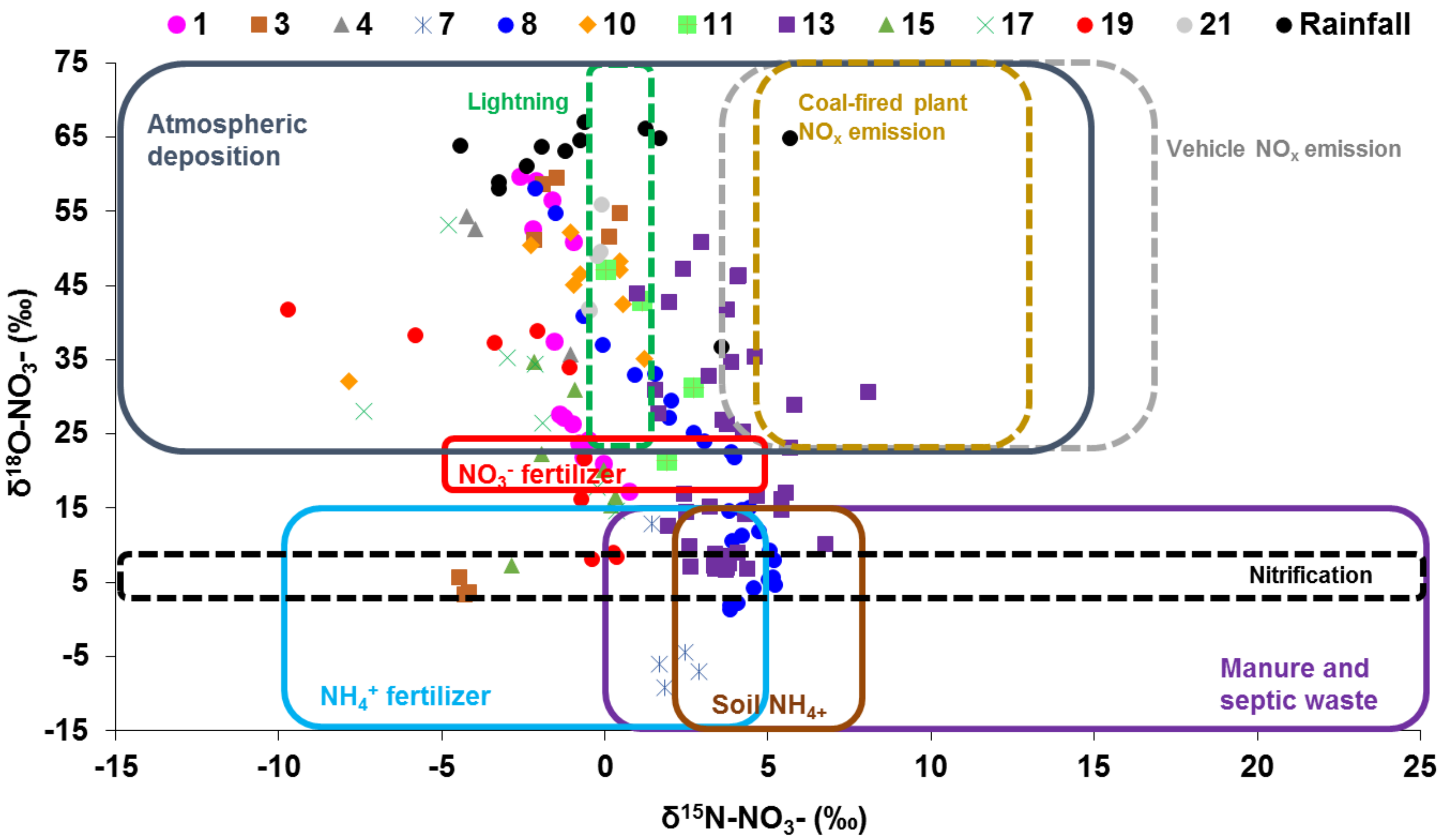

Fig 5. Stable isotopes of δ^{15}N-NO_3^- and δ^{18}O-NO_3^- in 12 sampled storm events from May to September 2016. Boxes indicate the range of the δ^{15}N–NO_3^- and δ^{18}O–NO_3^- values for NO_3^- sources according to (A) Kendall et al. (2007), Heaton (1990), and Felix et al. (2015) as shown in S2 Table.

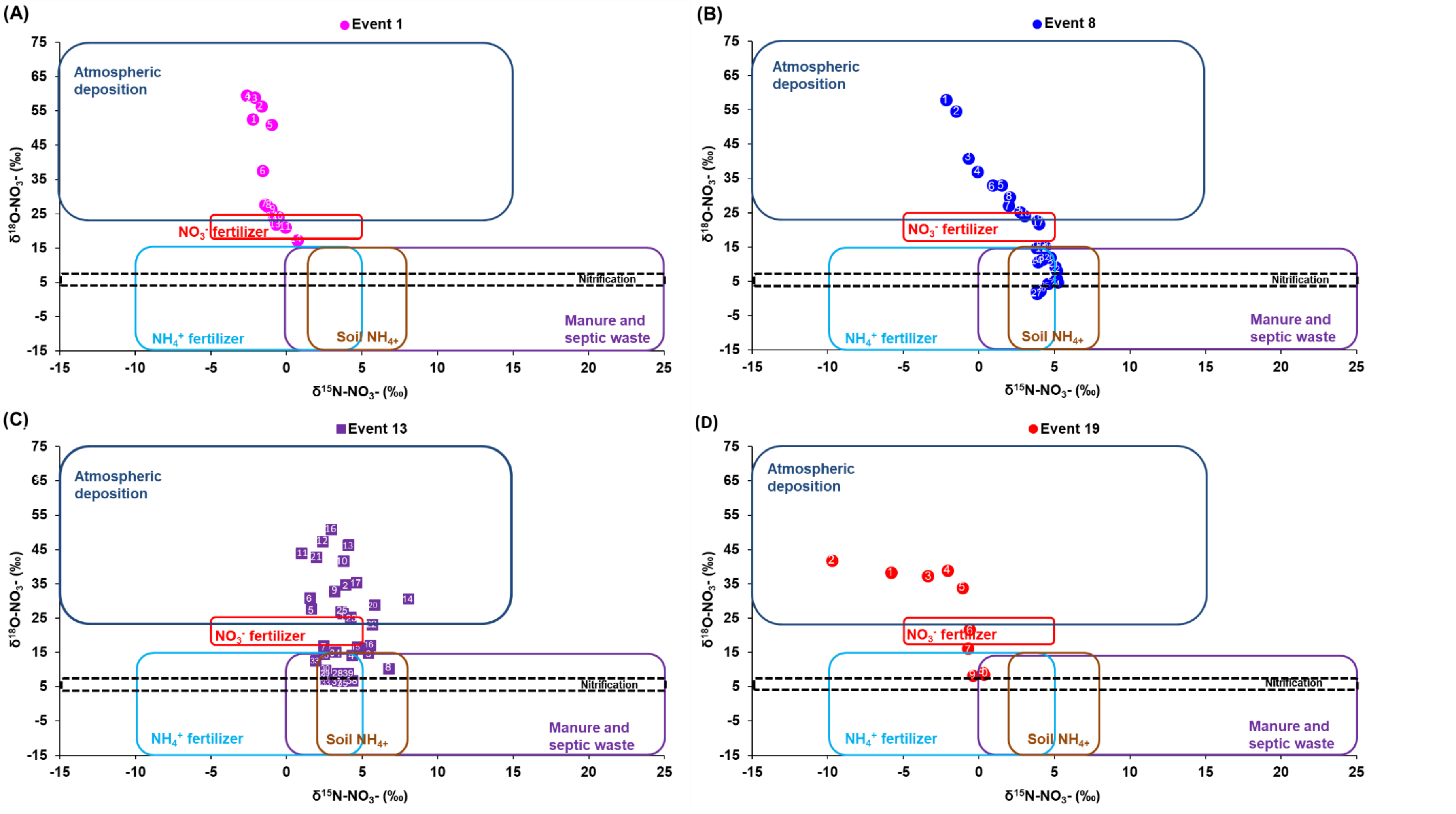

Fig 6. Stable isotopes of $\delta^{15}N–NO_3^-$ and $\delta^{18}O–NO_3^-$ in four individuals storm events (A) event 1 (n=14), (B) event 8 (n=27), (C) event 13 (n=39) and (D) event 19 (n=10) from May to September 2016.

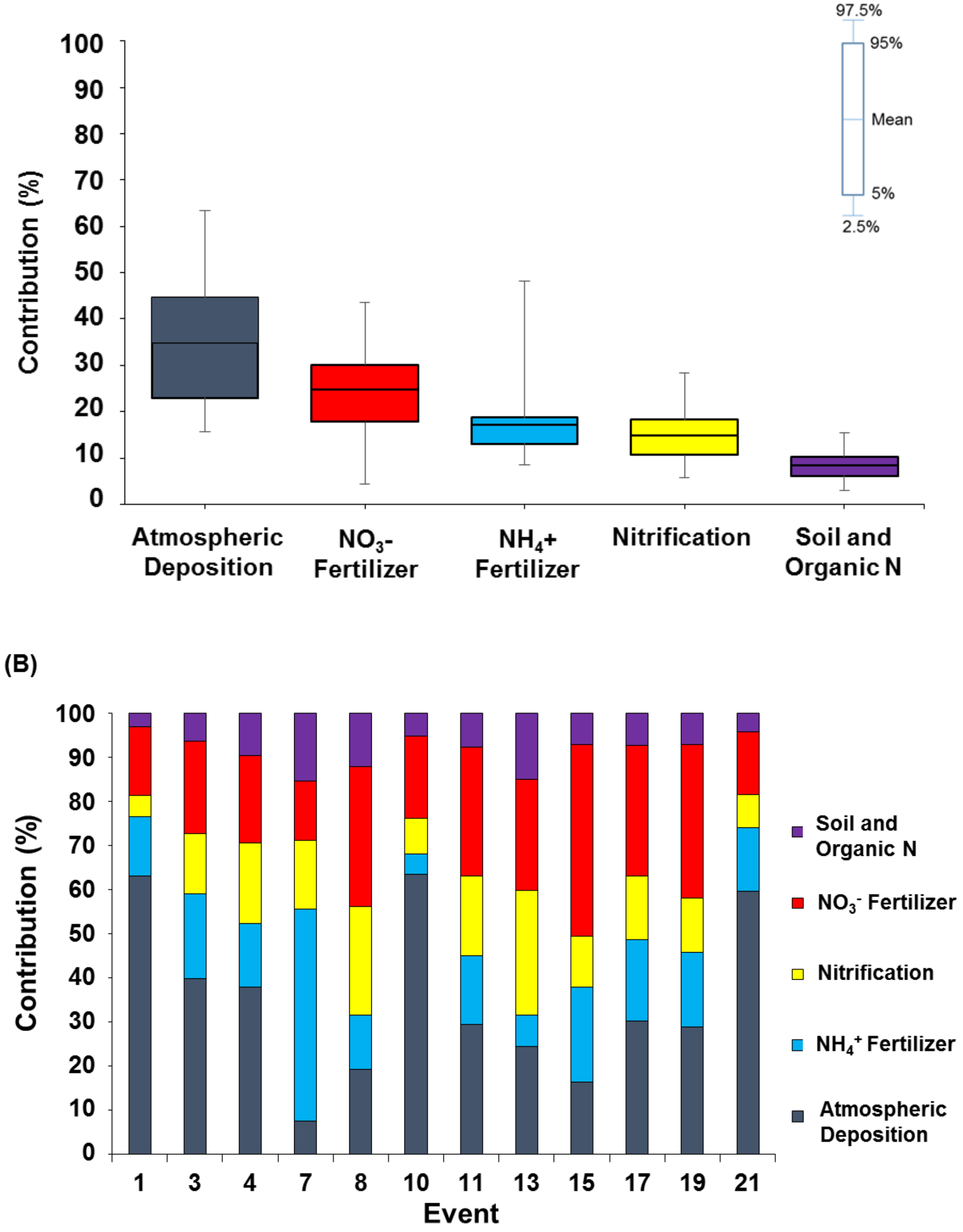

Fig 7. (A) Mean percent contribution of five nitrate sources from May to September 2016 and (B) Mean percent of NO_3^- sources for selected storm events (n=12) from May to September 2016.

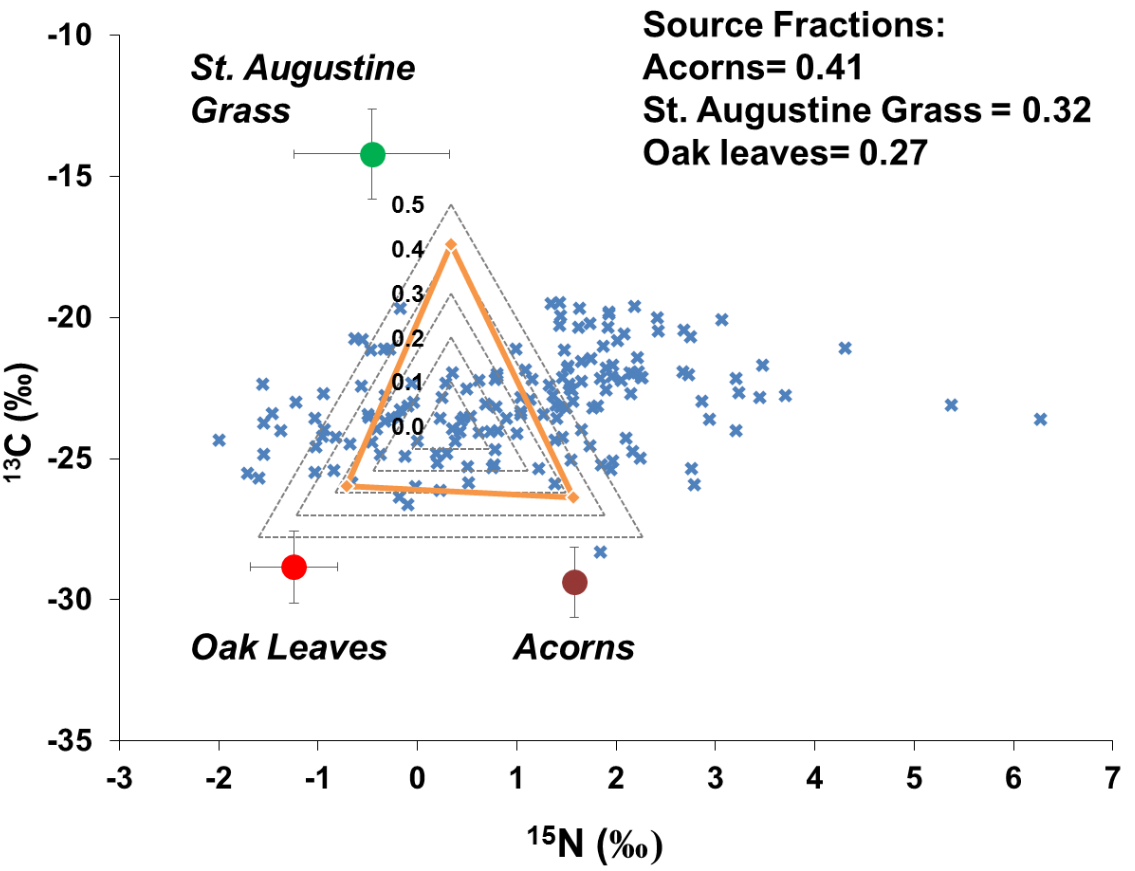

Fig 8. Values of δ^{15}N and δ^{13}C for endmembers (acorns, oak leaves, and St. Augustine grass) and runoff particulate organic N (PON) samples (blue crosses). Source proportions represent IsoError mixing model results and are estimates of the proportion of each source in the runoff samples.

Click here to access/download
Supporting Information
SI section.docx
