# Supplementary material for: Composition of nitrogen in urban residential stormwater runoff: Concentrations, loads, and source characterization of nitrate and organic nitrogen
Source: PLoS One. 2020 Feb 28;15(2):e0229715. doi: 10.1371/journal.pone.0229715 (PMC7048309; doi:10.1371/journal.pone.0229715)
Supplement: Attachment — Submitted filename: Responses-R1 .pdf [file pone.0229715.s013.pdf]

RESPONSE TO EDITOR AND REVIEWERS

RESPONSE TO THE EDITOR:

EDITOR COMMENT: *Your manuscript requires minor revisions based on the comments from three reviewers (all three agree that the manuscript requires minor revisions). Please ensure that in your revised manuscript you address all comments (note: one reviewer has provided comments directly on a PDF copy of the manuscript). I looked forward to the revised manuscript.*

RESPONSE: Thank you for coordinating the review process. We have revised the manuscript and addressed Reviewer's comments.

General Comments:

COMMENT: Please ensure that your manuscript meets PLOS ONE's style requirements, including those for file naming.

RESPONSE: **We have checked and attest that all formatting and style requirements have been met.**

COMMENT: In your Methods section, please provide additional information regarding the permits you obtained for the work. Please ensure you have included the full name of the authority that approved the field site access and, if no permits were required, a brief statement explaining why.

RESPONSE: **We added a sentence in "Study site instrumentation" section to indicate that we had permission from the Homeowners association to conduct research in the cathcment and that no permits were required.**

COMMENT: We note that Figure 1 in your submission contain [map/satellite] images which may be copyrighted. All PLOS content is published under the Creative Commons Attribution License (CC BY 4.0), which means that the manuscript, images, and Supporting Information files will be freely available online, and any third party is permitted to access, download, copy, distribute, and use these materials in any way, even commercially, with proper attribution. For these reasons, we cannot publish previously copyrighted maps or satellite images created using proprietary data, such as Google software (Google Maps, Street View, and Earth). For more information, see our copyright guidelines: http://journals.plos.org/plosone/s/licenses-and-copyright.We require you to either (1) present written permission from the copyright holder to publish these figures specifically under the CC BY 4.0 license, or (2) remove the figures from your submission:

RESPONSE: We have included information in the Fig caption that map was prepared using ArcGIS software.

RESPONSE TO REVIEWER 1:

REVIEWER COMMENT: *The manuscript represents a valuable addition to the literature on stormwater (SW) quality and general approaches to quality controls. While the initial approach to SW control focused on removals of suspended solids with attached chemicals, environmental concerns about nutrients exported with SW from urban catchments were addressed at a later date and often indicated transformations of various species of N and P in Best Management Practice facilities, or poor experimental methods and erroneous measurements. Hence, scientifically rigorous contributions, as the one reviewed herein, are useful precursors of further progress in this field. The manuscript is well thought out and written, the objectives clearly stated, experimental methods are advanced, data statistically analyzed, the conclusions well supported by the data presented, and in principle, the reviewer recommends its acceptance, pending some relatively minor corrections/clarifications improving the clarity of presentation. Speaking of clarity, the reviewer is slightly concerned that the authors depart in some cases from a common usage of hydrological terms and units, when describing the catchment hydrology (defined as those appearing e.g., in the Journal of Hydrology). I realize that these simple formal changes may involve enough work, and therefore, leave it up to the Editor to decide whether those changes are worthwhile. At the same time, the usage in the manuscript is somewhat distracting.*

RESPONSE: We thank the reviewer for kind words. We have revised our manuscript based on the comments as described below.

REVIEWER COMMENT: *Line (L) 66/67 – flow (presuming runoff flow) should not be listed as a rainfall variable.*

RESPONSE: We have removed "flow" from the sentence.

REVIEWER COMMENT: *L101 – runoff volume is not a rainfall variable*

RESPONSE: We have removed "runoff volume" from the sentence.

REVIEWER COMMENT: *L107-123 – the study catchment description is incomplete; for applications elsewhere, one would be interested e.g. in an average lot size; whether the impervious areas are directly connected to sewers, or some of them discharge on pervious parts of the catchment; do roofs discharge onto lots, or are they connected to sewers?; soil type (and even just a qualitative description of soil infiltration); whether any runoff controls are used on individual lots, or in the catchment.*

RESPONSE: We have expanded the description of the study catchment to include information about how runoff from the catchment flows to a stormwater retention pond structure, as well as information about soils, infiltration, and other runoff control structures in "Site description" section.

REVIEWER COMMENT: *L116 – surface runoff does not enter into curbs, but street gutters and then into storm sewers (or drains) before draining…; any comments on the N fertilizer ban compliance?*

RESPONSE: The sentence has been revised. The community practiced the N fertilizer ban during the rainy season from June to September 30, this is discussed in Results and Discussions section.

REVIEWER COMMENT: *L124 – annual precipitation and air temperatures, any other comments re the catchment climate.*

RESPONSE: We have added a few sentences regarding annual precipitation and temperature. S1B Fig has more data on rainfall.

REVIEWER COMMENT: *L126 – the rain gauge is not installed in the storm sewer outlet, but at, or by; and incidentally, the one shown in Fig. 1 is most likely "shaded" by the nearby trees (bias in in rainfall capture) and measurement accuracy. How were the rainfall events defined? They are usually defined by the minimum rainfall depth (e.g., > 2 mm) and minimum inter-event time (e.g., 6 hours) – needed to distinguish between events with shorter periods of no rain and two events generated by different whether systems.*

RESPONSE: The rain gauge was installed close to the storm sewer inlet and not shaded by the nearby trees (there are not any big trees to shade the area where rain gauge was installed). While all rain events were recorded, we collected runoff only from those that met a threshold value of 2.5 mm of rainfall for 10 minutes or a minimum level of 19.1 mm for 5 minutes in the inflow pipe. This information is included in the "Study site instrumentation" section.

REVIEWER COMMENT: *L130-132 – flow monitoring – the model number?, some estimates of accuracy?*

RESPONSE: The flowmeter is 360 LaserFlow Non-contact Velocity Sensor. The accuracy level has been added in "Study site instrumentation" section.

REVIEWER COMMENT: *L163-167 – please check eq. (2-1) – I believe that it is incorrect (assuming that n is a sample count); as it is written, I can cancel "ti*ci" in the numerator and denominator and the equation then reads FWMC = sum (ci), from 1 to n.*

RESPONSE: We have corrected and revised the eq. (2-1).

REVIEWER COMMENT: *L170 – rainfall samples – I do not recall reading about them earlier, how and where they were collected?*

RESPONSE: Rainfall samples were collected from the sample bottles connected to the rain gauge. Information has been added to the manuscript in "Sample collection and preparation" section.

REVIEWER COMMENT: *L221 – the rainfall variables are listed correctly (contrarily to Line 101); rainfall "amount" is generally measured as rainfall depth [mm]; the intensity – is that event mean intensity?*

RESPONSE: Yes, it is the event mean intensity.

REVIEWER COMMENT: *L226 and elsewhere – total rainfall of 1,055 mm (this applies throughout the manuscript)*

RESPONSE: Changes have been made throughout the manuscript.

REVIEWER COMMENT: *L232 – 233 – two decimal points 2.54 mm, 19.05 mm is excessive and misleading as to measurement accuracy, use 2.5 and 19.1 mm; …2.5 mm of rainfall in 10 minutes…; …the in the…, L233, delete the first the.*

RESPONSE: We have kept to two decimal places as this is how the instruments were set up by a trained technician from the manufacturer.

REVIEWER COMMENT: *L236 – the rainfall intensity …ranged from 2.0 to 5.1 mm hr-1 …. – the intensities were averaged over the storm event duration; for later discussion of N export from the catchment, peak intensities over some fixed time interval (close to the catchment time of concentration, perhaps 15 – 30 minutes, would be more relevant.*

RESPONSE: As some events lasted for <30 minutes, it is better to keep these units to an hour. Secondly, most papers in literature report intensity per hour, therefore, it is better to keep the units as such to allow comparison by other authors to our study.

REVIEWER COMMENT: *L237-238 – unusual units of total volume (rather than amount) of runoff – the larger number equals 554 mm of runoff and the smaller 168 mm of runoff (from the catchment studied). 1 litre is perhaps much too small unit of volume; why not m3 ?*

RESPONSE: We agree with the Reviewer. We now have revised the units to m^3 in the manuscript and figures (e.g., Fig 2).

REVIEWER COMMENT: *L276-277 – it would be useful to define "high" rainfall and runoff quantitatively*

RESPONSE: Values have been added into the sentence to define 'high' runoff.

REVIEWER COMMENT: *L281-284 – this has been defined in the literature as "pollutant build-up and wash-off" (e.g., in the US EPA SWMM model, or see e.g. Vaze, J. and Chiew, H.S. (2003). Study of pollutant washoff from small impervious experimental plots. Wat.Res. Research, 39(6), 1160; doi: 10.1029/2002WR001786*

RESPONSE: We have added a few sentences on this and included the reference provided by the reviewer.

REVIEWER COMMENT: *L302 – rainfall amount listed twice*

RESPONSE: The sentence has been revised.

REVIEWER COMMENT: *L383 - …winter season, reaction occurs….*

RESPONSE: Correction has been made.

REVIEWER COMMENT: *L496 – 497 – it is not just the issue of age of sanitary and storm sewers, but more importantly, no cross-connections (misconnections) between both systems.*

RESPONSE: We agree with the reviewer and have made the revision.

REVIEWER COMMENT: *L521-523 – it is hard to visualize "rotting" leaves and acorns in street gutters in an obviously upscale residential area; does this really happen?*

RESPONSE: Yes, we did observe decomposed materials near the gutter during our study period as many leaves and plant material usually hang in the gutters till they are flushed.

REVIEWER COMMENT: *L542 – perhaps "longer antecedent dry season"*

RESPONSE: We have revised the sentence.

REVIEWER COMMENT: *L556 – it is easy and "logical" to suggest cleaning streets before storm events, but that is a very costly proposition. Perhaps one should somehow temper this recommendation. Also, there is a stormwater pond designed to control runoff from the study area, does it help the N situation? (I know this is outside of the paper scope, but so are your comments on SW quality controls).*

RESPONSE: We agree with the reviewer. We now have added a few sentences.

RESPONSE TO REVIEWER 2:

REVIEWER COMMENT: *This article is well written and provides an interesting assessment of nitrogen species partitioning in urban runoff. The most compelling section was the isotope analysis based on its novelty. I have relatively minor comments on the attached document. I have a few items that should be cleared up for publication. In particular, I would ask the authors to specify if they checked for normality of the data.*

RESPONSE: We thank the Reviewer for kind words. We have revised our manuscript based on the Reviewer's comments as below. We also confirm that we did check for and observe normality of the data.

REVIEWER COMMENT: Line 121: Make this link a reference instead - as in, show the link in the references and cite here as appropriate

RESPONSE: We have removed the hyperlink from the manuscript body.

REVIEWER COMMENT: line 154: you were able to analyze unfiltered samples in this machine?

RESPONSE: We had this concern that it would not be possible to run samples for TN and these samples would ruin the instrument, however, the runoff waters in this catchment were very clear and clean (no color or sediments), Thus, we were able to run the samples directly and unfiltered, which saved some analysis time.

REVIEWER COMMENT: Line 267, maybe also comment on how these concentrations compare to other studies.

RESPONSE: We have expanded the discussion to include how these concentrations compare to other studies including our work in Florida.

REVIEWER COMMENT: LINE 412, You cite Baral et al. from Journal of Env Eng. - didn't they show this too?

RESPONSE: We agree with the reviewer and have edited this sentence to take out the part about our study being the first to see evidence of fertilizer mobilization.

RESPONSE TO REVIEWER 3:

REVIEWER COMMENT: *The authors present an extensive and very valuable stormwater data set; in particular, the within-event isotopic analyses are a unique and interesting data set. The data have been thoroughly checked against previous studies and data sets. The field, lab, and statistical analyses are solid, and appropriate for the work. The paper is also pretty well written, logically organized, and thoroughly referenced. The results are a bit long and the conclusions a bit light, but mostly appropriate given the large size of the data set.*

I have a few general comments that could potentially improve the clarity and impact of the results

RESPONSE: We thank the reviewer for kind words. We have revised our manuscript based on the comments as described below.

REVIEWER COMMENT: *I think the conclusions could be strengthened by digging into the more novel aspects of the data set (DON, PON sources; within-event source dynamics) and making recommendations for BMPs or providing support for existing BMPs for treating those forms of N.*

RESPONSE: We have included a few sentences and modified existing sentences to strengthen the conclusions section.

REVIEWER COMMENT: *There is a lot of detail on the NO3 results (for good reason, given the extensive number of isotope samples that were processed), but it appears to be a minor component of TN at this site, and not a lot to be concluded from the results. NO3 and NH4 concentrations of 0.2mg/L (mean) are pretty low. I think the organic N part of the work is potentially more useful / interesting.*

RESPONSE: We agree and have added discussion in the conclusions to draw out the importance of the organic N component. We assert, however, that the NO_3 data is worthwhile considering our study location and current (very controversial) urban fertilizer legislation that puts a strong focus on inorganic N mobilization via stormwater. We have included a brief discussion of this in the beginning of the section on nitrate sources and in the conclusion.

REVIEWER COMMENT: *I would be interested in learning more about the within-event concentrations and isotopic composition, and what the results might mean for type or timing of stormwater management; and how might event characteristics (intensity, depth, duration), which vary across climates, impact prescribed management? I feel that this is a very important component of your data set!*

RESPONSE: Great point that we whole-heartedly agree, however, there is tremendous complexity and variability and thus it is impossible to draw and extend conclusions from one event to others. Several parts of the manuscript do address this issue. For example, Fig 6 and section "Changing sources of nitrate in storm events" and S4 Figure and S5 Figure and their discussion in the manuscript highlight the issue of variability within events. As more studies are conducted worldwide, we believe it would allow us to better understand and perhaps develop solutions for stormwater management that are more site-specific and focused on individual events rather than over seasons.

REVIEWER COMMENT: *"percent runoff" / "percent of runoff" needs to be more carefully defined for clarity (see comment below)*

RESPONSE: We agree with the Reviewer. We now have changed it to percent runoff throughout the manuscript.

Reviewer Comment: Minor Comments - check references to tables/figures so that they are consistent with the form required by the journal, e.g. "S4 Table" in L276 (should this be "Table S4?"), "Fig 3A and 4B" in L278 (should this be "Figs. 3A and 4B"), and elsewhere

RESPONSE: S4 Table is based on the format required by the journal. We have now revised Figs 3A and 4B and others through the manuscript.

Reviewer Comment: in citing studies, be consistent with locations (i.e. use City, State, Coutry or just State, Country)

RESPONSE: We have revised the citing studies in the manuscript.

Reviewer Comment: Methods - does the site have any baseflow? threshold for sampling seems small so guessing not but maybe good to mention

RESPONSE: There is no baseflow in this urban residential site. We now have added a sentence on this.
Reviewer Comment: L135-144: check grammar (“enabled to monitor”… etc)

RESPONSE: We have revised the sentence.

Reviewer Comment: L146+: time paced sampling, or flow paced sampling? FWMC implies time pacing but is unclear.

RESPONSE: The criteria for sampling is included in the method section. It was time-paced sampling (samples were collected every 10 minutes).

Reviewer Comment: each sample bottle analyzed separately, right?

RESPONSE: Yes, that’s correct.

Reviewer Comment: L160: is FWMC calculated for each event, or across all samples? If this is calculated across all samples, this would seem to bias results towards more frequently sampled storms (?)

RESPONSE: We calculated FWMC for each individual sample and event.

Reviewer Comment: L222: omit comma

RESPONSE: We have revised the sentence.

Reviewer Comment: Results: L233, L238: seems like a small threshold for runoff yet only 36% of rainfall and 30% of runoff was sampled — how applicable might results be for small storms?

RESPONSE: Small (<2.5 mm) rainfall events are common in our study site. These small storms rarely generate runoff because rainfall at such small depths is quickly infiltrated by sandy soils or evaporates before building up enough volume to generate runoff on impervious surfaces. Due to the sandy soils and hot summer climate, the catchment dries out quickly between these small, frequent rainfall events. In addition, there are also practical logistic issues such as instrument not able to take samples (battery issues). We believe that capturing about 1/3 of rainfall and runoff is pretty good in urban catchment as this provide good information about variability that could be expected in runoff chemistry.

Reviewer Comment: L242: what quantities are being compared here — volumes? And if so, how is this different from the result presented in L248-9 (and Fig 3C)? Also, does the correlation include sampled events, or all events? Correlation of rainfall and runoff is an intuitive/obvious result, but this still needs to be explained.

RESPONSE: Yes, runoff volumes. The correlation was done by all 75 events. We have revised the sentence.

Reviewer Comment: L242-3 (and L332): what is "percent runoff" — fraction of rainfall that became runoff, or fraction of total runoff that was sampled, or something else? The first definition is implied by the subsequent lines, but an explicit definition would be helpful. This is also especially confusing because the y-axis label in Fig 3A (and x-axis in 3B) is "percent OF runoff", which implies a potentially different definition (e.g. "percent of runoff that was observed", rather than "percent of rainfall as runoff"). I think this term needs to be changed for clarity, even if is more verbose.

RESPONSE: Thank you for this comment. We meant to say "percent runoff—fraction of rainfall that becomes runoff and have changed the text and axes labels in Fig 3 accordingly.

Reviewer Comment: L247: similarly, 10-80% of what? rainfall?

RESPONSE: 10-80% is percent runoff (of total rainfall) during the wet season. We have revised the sentence.

Reviewer Comment: L260: should be "Fig. S2"?

RESPONSE: According to the journal formatting. It should be S2 Fig.

Reviewer Comment: L282-4: check grammar

RESPONSE: We have revised and clarified the grammar of this sentence.

Reviewer Comment: L333: course should be coarse

RESPONSE: We have revised the word.

Reviewer Comment: L342: are these expected to be major sources of water in your study area?

RESPONSE: In our study, the runoff waters captured originates with rainfall. Some other sources of runoff could be irrigation water and reclaimed water (if used) for irrigation in particular. In our study site, no reclaimed water was used.

Reviewer Comment: L363-4: this seems to conflict with the last sentence of the previous paragraph, which stated that rainfall and runoff samples were "identical with each other" — please clarify what difference is being referenced here (event-to-event perhaps?)

RESPONSE: We now have revised the sentences.

Reviewer Comment: L370+, L352+, L501+: would it be useful to condense the isotope data (means and ranges) to a table, maybe along with references for similar studies? Your main points in these paragraphs (e.g. that runoff water was nearly all rainfall, explanation of seasonal and

intra-event variability of dN-NO3) get buried at the end of the paragraphs. I think these would be easier to read and stronger paragraphs if you lead each with a main result from your study. Might be personal preference, though.

RESPONSE: We agree with the Reviewer. We now have added the means and ranges of isotope data into the S4 Table.

Reviewer Comment: L393-395: this is a somewhat vague explanation of variability of isotopic composition within events — you provide a nice explanation in the previous paragraph for seasonal variability of dN-NO3, but perhaps you could do more with your data to explain how the changes in this isotope over an event explain changes in N source?

RESPONSE: We have revised the sentences to facilitate better reading and understanding of this section.

Reviewer Comment: L397: I think this section could be expanded a bit to discuss changing sources during events, and if possible, discussing both NO3 and ON.

RESPONSE: We have included some discussion regarding source changes during events in this section.

Reviewer Comment: L403: the result beginning on L403 (change in source over the event) seems like a more interesting starting point than just summarizing your isotope results. Or could start a new paragraph here to discuss changes in source over an event. This is an important issue that your dataset is well equipped to address!

RESPONSE: We agree with the Reviewer. We now have started a new paragraph.

Reviewer Comment: L410: this sentence needs clarification. Why would DIN become a larger component of TN as the larger events become "enriched" in ON? This would imply the opposite pattern. Do you perhaps mean "saturated" in ON, i.e. only so much can be mobilized?

RESPONSE: We agree this sentence was confusing and it did add anything to the discussion. We removed this sentence.

Reviewer Comment: 413: Discussion of fertilizer kind of comes out of nowhere. Might be good here to point readers to Fig 5 again to show the importance of fertilizer — which appears to be important only for a few events?

RESPONSE: We agree with the Reviewer. We have now cited Fig 5 in the sentence.

Reviewer Comment: 424-499: I realize that the substantial amount of nitrate isotope data collected compels you to do a source-tracking analysis, which is interesting, but it comprises such a small component of stormwater N at your site, and the largest component is atmospheric, such that management implications are pretty minimal. The thorough discussion of the various components of atmospheric NO3 (L450-466) and organic NO3 (L481-99) do not add much to

the main points of your paper and could potentially be condensed into a single paragraph discussing other NO3 sources. - this section also needs to reference Fig 6

RESPONSE: We agree and have shortened this section, with a new emphasis on the fertilizer contributions. We have also referenced Figure 6 in this discussion.

Reviewer Comment: L467+: I think the fertilizer section might be the most important part of the NO3 results, especially in light of the fertilizer ban in place during the study period. How does the percentage of NO3/NH4 as fertilizer in your study compare to the other cited studies? If fertilizer restrictions were NOT in place in those other studies, then you have an interesting result for the effectiveness (or lack thereof) of a fertilizer ban.

RESPONSE: We have shortened this section with a new emphasis on the fertilizer data.

Reviewer Comment: L510, L517: this is an important result. It might be personal preference, but leading with a summary of the results is less exciting than leading with your major outcome. Also, oaks may be considered "messy" trees; how might the results change with a watershed containing different deciduous species (or mix of species)?

RESPONSE: We have revised the sentence and moved the summary at end of the paragraph. Oaks are the most abundant trees in Florida landscapes. We do not have information about other trees and how that will change the composition. This is also beyond the scope of this study.

Reviewer Comment: L520: Ong reference needs to be in numbered form.

RESPONSE: We have revised the text.

Reviewer Comment: Conclusions - Mostly just a summary of the results. What have you learned from the data set? (Most of the BMP suggestions in the conclusions are practices that are already being implemented for both N and P management.) For example, what insights did you gain from doing the within-event analyses, which are fairly rare as a data set?

RESPONSE: we have added several sentences to better frame the context and conclusions section.

Reviewer Comment: L537: if DON is the dominant form, what might be some management options? The BMPs suggested for management of NO3 (L547-9), which is a minor component of TN, are already pretty standard practices, so this is not new information.

RESPONSE: As you might be aware, it is possible to control DON. This requires holistic evaluation of the sources of the DON and what could be done to prevent DON losses. We have included a sentence on this in Conclusions section.

Reviewer Comment: L554+: does this imply that street sweeping could be a particularly effective BMP?

RESPONSE: Previous studies have suggested street sweeping can be an effective strategy for reductions of TN load (e.g., Selbig W. 2016. Sci. Total Environ. 571: 124-133; Baker et al. 2014. Stormwater. 15: 16-23).

Reviewer Comment: Fig 2 - line graph implies continuity from point to point, yet these are discontinuous data (discrete x axis rather than continuous/time). Could this info be condensed into a table, or perhaps make all of the plots bar graphs (like top plot in figure)?

RESPONSE: We agree with the Reviewer. We now have revised Fig 2 based on the suggestion.

Reviewer Comment: Fig 3 - A, B: see above concerns about "percent of runoff" label on y-axis.

RESPONSE: We now have revised the y-axis of Fig 3 to percent runoff.

Reviewer Comment: Fig 3 - C: is the line fit to all events or just sampled events?

RESPONSE: Yes, the line fits to all events. We now have indicated that line fit is for sampled events in the legend.

Reviewer Comment: Fig 4 - B: y-axis is really hard to read; possible to make text larger?

RESPONSE: We agree and have revised Fig 4 for a better quality.

Reviewer Comment: Fig 4 - legend: remove "for easy visualization" (which is subjective) and describe how they are grouped — it appears they are grouped by magnitude of TN (i.e. scale)?

RESPONSE: We have now revised the legend based on the suggestion.

Reviewer Comment: Fig 6- Legend: please give the dates of the events (rather than "May to September 2016"), and maybe rainfall amounts? Also please explain that "n" is number of samples within the event.

RESPONSE: We now have revised Fig 6 based on the suggestions.
